# Curvature Templates in Clinical Information Geometry: Fisher Manifolds, Relational Embeddings, and Worked Examples

**DOI:** 10.3390/e28080868

**Published:** 2026-08-01

**Authors:** Dragoş Petru Teodor Iancu, Călin Gheorghe Buzea, Florin Nedeff, Diana Mirilă, Valentin Nedeff, Mirela Panainte-Lehaduș, Claudia Manuela Tomozei, Maricel Agop, Pintilie Tudor Florin, Roxana Irina Iancu, Lăcrămioara Ochiuz, Decebal Vasincu

**Affiliations:** 1 Department of Oral Pathology, “Grigore T. Popa” University of Medicine and Pharmacy, 700115 Iaşi, Romania; dt_iancu@yahoo.com; 2 Department of Oncology and Radiotherapy, “Grigore T. Popa” University of Medicine and Pharmacy, 700115 Iaşi, Romania; 3 Department of Radiotherapy, Regional Institute of Oncology, 700483 Iași, Romania; 4 National Institute of Research and Development for Technical Physics—IFT Iași, 700050 Iași, Romania; calinb2003@yahoo.com; 5 Clinical Emergency Hospital “Prof. Dr. Nicolae Oblu” Iași, 700309 Iași, Romania; 6 Department of Environmental Engineering, Mechanical Engineering and Agritourism, Faculty of Engineering, “Vasile Alecsandri” University of Bacău, 600115 Bacău, Romania; florin_nedeff@ub.ro (F.N.); vnedeff@ub.ro (V.N.); mirelap@ub.ro (M.P.-L.); claudia.tomozei@ub.ro (C.M.T.); m.agop@yahoo.com (M.A.); 7 Faculty of Physics, Alexandru Ioan Cuza University of Iași, 700506 Iași, Romania; tudorflorinpintilie@gmail.com; 8 Department of Clinical Laboratory, “St. Spiridon” Emergency Hospital, 700111 Iași, Romania; 9 Faculty of Medicine, “Grigore T. Popa” University of Medicine and Pharmacy, 700115 Iași, Romania; lacramioara.ochiuz@umfiasi.ro (L.O.); decebal.vasincu@umfiasi.ro (D.V.)

**Keywords:** Fisher information geometry, clinical state spaces, curvature templates, covariance-based diagnostics, relational graphs, compositional data

## Abstract

Clinical prediction models commonly represent patients as points in high-dimensional feature spaces, but they rarely examine the information geometry generated by the statistical organization of clinical states. This study develops a theoretical and computational framework in which clinical states are represented as coarse-grained relational ensembles and analyzed through Fisher information geometry. We use two established statistical manifolds as controlled curvature templates: the Gaussian location–scale family, which has negative Fisher scalar curvature and represents fluctuation-dominated organization, and the categorical/simplex family, which has positive Fisher scalar curvature and represents normalized finite-capacity organization. The main contribution is the clinical-relational operationalization of these templates, together with worked examples showing how candidate clinical observables may be mapped to fluctuation-dominated, compositional, and graph-based settings. We further distinguish exact Fisher-geometric results from covariance-based diagnostic proxies used in finite simulations, and we introduce a reproducible protocol including metric-conditioning checks, sensitivity analysis, and a Riemannian–Laplace approximation for higher-dimensional tractability. The framework is not presented as a validated diagnostic or prognostic model. Rather, it provides a hypothesis-generating geometric layer for studying instability, constraint, and state-space organization in complex clinical systems.

## 1. Introduction

Clinical prediction models increasingly rely on high-dimensional representations of patients, including laboratory measurements, physiological time series, imaging features, molecular profiles, treatment variables, and longitudinal trajectories. In most statistical and machine learning approaches, these quantities are treated as features in a predictive space, and the patient is represented as a point, vector, or trajectory in that space. This representation is useful and has supported major progress in risk prediction, radiomics, intensive-care modeling, and precision medicine. However, it does not directly describe the intrinsic statistical geometry generated by the organization of clinical states. In particular, it does not ask whether nearby disease states are statistically distinguishable in the same way across all regions of the state space, or whether some regions are organized by instability, fluctuation, constraint, or compositional balance.

Information geometry provides a natural language for this problem. In a statistical model, the Fisher information metric defines a Riemannian geometry on the parameter space by measuring the local distinguishability of probability distributions [1,2,3,4,5]. From this perspective, a clinical state is not only a vector of observed variables but may also be interpreted as a probability distribution or an ensemble of possible relational configurations. Distances, curvature, and metric conditioning then describe properties of statistical distinguishability rather than anatomical distance or physical spacetime structure. This study uses this restricted Fisher-geometric meaning of curvature throughout.

This distinction is important because complex disease states are rarely organized by independent variables alone. Critical illness, cancer progression, inflammatory response, treatment response, recurrence, and recovery involve interacting physiological, molecular, imaging, and temporal components. Network medicine has emphasized that disease phenotypes can be understood through relational structures among biological and clinical variables [6]. Compositional data analysis has shown that normalized fractions, such as cell-type proportions or compartmental distributions, require a dedicated geometry because the components are constrained by a fixed total [7]. Entropy-based analysis of physiological time series and radiomics further illustrates that clinically relevant information may reside in variability, heterogeneity, and multiscale organization rather than in isolated measurements [8,9,10].

This study addresses a specific methodological question: whether selected families of clinical observables can be represented by statistical manifolds whose Fisher geometry has a controlled curvature sign. Two established information-geometric examples are used as mathematical templates. The first is the Gaussian location–scale family, whose Fisher geometry is hyperbolic and has negative scalar curvature. In the clinical interpretation proposed here, this template represents fluctuation-dominated organization, where an observable has both a mean level and an unconstrained variability or instability scale. The second is the categorical probability simplex, whose Fisher geometry is locally spherical and has positive scalar curvature. In the proposed clinical interpretation, this template represents normalized finite-capacity organization, where components compete within a bounded total, as in compositional biomarkers, immune-cell fractions, tumor-subregion proportions, or trajectory-motif fractions [3,4,5,7].

The mathematical curvature of these two statistical manifolds is not claimed as a new result. The contribution of this study is to use these known Fisher-geometric structures as controlled templates for clinical state organization and to show how they may be operationalized in a relational clinical setting. Specifically, the study separates established information-geometric results from the proposed clinical interpretation and from the computational diagnostic procedures used in finite simulations. This distinction is essential because a negative-curvature Fisher manifold does not automatically imply clinical deterioration, and a positive-curvature simplex does not automatically imply clinical stability. Rather, curvature sign becomes clinically interpretable only after the observables, statistical family, coordinate system, and normalization constraints have been explicitly specified.

To move from interpretation to implementation, three worked examples are considered. A fluctuation-dominated example illustrates how a physiological or imaging-derived observable with a mean and variability scale can be mapped to a Gaussian location–scale template. A normalized finite-capacity example illustrates how motif fractions or compartment proportions can be mapped to a simplex template. A graph-based relational example then shows how nodes, edges, weights, coarse-grained observables, and covariance-based diagnostic proxies can be defined in a reproducible way. This relational construction builds on the axiomatic relational–informational framework previously proposed for emergent geometry and effective spacetime but adapts it here to clinical statistical states rather than to physical spacetime structure [11]. These examples are not presented as validated diagnostic models, but as methodological demonstrations of how curvature analysis could be applied to clinical data structures.

Because exact Fisher-metric reconstruction becomes difficult in high-dimensional or non-closed-form settings, a tractability layer is also required. In analytic template models, curvature can be computed directly from the Fisher metric. In finite graph simulations, however, empirical covariance quantities are used only as Fisher-consistent diagnostic proxies unless a natural-parameter map or coordinate pullback is explicitly specified. For higher-dimensional applications, a Riemannian–Laplace approximation provides a practical local approximation strategy around dominant macrostates while preserving the geometric role of the metric [12,13]. This distinction clarifies the computational status of the proposed framework and avoids overstating what can be inferred from finite numerical proxies.

The aim of the paper is therefore not to replace conventional statistical or machine learning models, nor to claim clinical validation of curvature biomarkers. Rather, the aim is to provide a geometric layer for hypothesis generation: fluctuation-dominated observables may be studied through negative-curvature Fisher templates, normalized finite-capacity observables through positive-curvature simplex templates, and relational clinical systems through graph-based diagnostic embeddings. Future validation requires disease-specific datasets, explicit observable definitions, reproducible graph construction, and independent testing of whether curvature-derived quantities add information beyond conventional statistical and machine learning models.

## 2. Model and Computational Framework

### 2.1. Clinical Statistical States and Fisher Information Geometry

Let *x* denote an observed clinical quantity or a vector of clinical observables. Depending on the application, *x* may represent a physiological measurement, a laboratory marker, an imaging-derived feature, a radiomic descriptor, a graph-derived observable, or a coarse-grained trajectory statistic. A family of clinical statistical states is represented by a parametric model:

(1) $$p\left(x|\theta \right)$$

where $\theta =\left(\theta_{1},\theta_{2},\ldots ,\theta_{d}\right)$ denotes a set of macroscopic parameters describing the clinical state.

The Fisher information metric associated with this family is defined as

(2) $$g_{ij}\left(\theta \right)=E_{\theta }\left[\frac{\partial \log p\left(X|\theta \right)}{\partial \theta_{i}}\frac{\partial \log p\left(X|\theta \right)}{\partial \theta_{j}}\right]$$

Here, $g_{ij}\left(\theta \right)$ is the (*i*, *j*) component of the Fisher information metric, and the expectation is taken with respect to the probability distribution $p\left(X|\theta \right)$.

Under standard regularity assumptions, the Fisher metric may also be written as

(3) $$g_{ij}\left(\theta \right)=-E_{\theta }\left[\frac{\partial^{2}\log p\left(X|\theta \right)}{\partial \theta_{i}\partial \theta_{j}}\right]$$

The corresponding line element on the statistical manifold is

(4) $$ds^{2}=\sum_{i=1}^{d}\sum_{j=1}^{d}g_{ij}\left(\theta \right)d\theta_{i}d\theta_{j}$$

This metric measures the local statistical distinguishability of nearby clinical states. Two parameter values are close when the corresponding probability distributions are difficult to distinguish statistically. They are far apart when small changes in *θ* produce clearly distinguishable distributions. In this sense, the geometry considered here is not anatomical, spatial, or gravitational. It is the geometry of statistical distinguishability.

The scalar curvature *R*(*θ*) is computed from the Fisher metric and its first and second derivatives using the standard Riemannian construction. In two dimensions, scalar curvature is related to Gaussian curvature *K* by

(5) R=2K

Throughout this study, curvature sign is interpreted only within this Fisher-geometric meaning. A negative value of *R* indicates a hyperbolic-type statistical geometry, whereas a positive value indicates a spherical-type statistical geometry. The clinical interpretation of these signs depends on the observable family used to construct the statistical manifold.

### 2.2. Established Curvature Templates

The framework uses two established two-dimensional Fisher manifolds as controlled templates. They are not introduced as new mathematical results. Their role is to provide analytically tractable reference geometries for two different types of clinical organization.

The first template is the Gaussian location–scale family,

(6) $$p\left(x|\mu ,\sigma \right)=\frac{1}{\sqrt{2\pi }\sigma }\exp\left[-\frac{{\left(x-\mu \right)}^{2}}{2\sigma^{2}}\right],\;\sigma >0$$

Here, *μ* is the location parameter and *σ* is the scale parameter. In the clinical interpretation, *μ* represents the average level of a clinical observable, while *σ* represents its fluctuation amplitude, dispersion, or instability scale.

For this family, the Fisher metric in coordinates (*μ*, *σ*) has the components

(7) $$g_{\mu \mu }=\frac{1}{\sigma^{2}}$$

(8) $$g_{\mu \sigma }=0$$

and

(9) $$g_{\sigma \sigma }=\frac{2}{\sigma^{2}}$$

Equivalently, the line element is

(10) $$ds^{2}=\frac{1}{\sigma^{2}}d\mu^{2}+\frac{2}{\sigma^{2}}d\sigma^{2}$$

The corresponding Fisher manifold has constant negative scalar curvature:

(11) $$R_{Gaussian}=-1$$

A complete derivation of the Fisher information metric and scalar curvature for the Gaussian location–scale family is provided in Appendix A.

This template represents statistical families in which both the average level and the variability scale of an observable are relevant. In the clinical interpretation proposed here, this structure is used as a sufficient template for fluctuation-dominated organization. Examples include observables whose clinical meaning depends on variability, dispersion, heterogeneity, or instability scale.

The second template is the three-state categorical probability simplex. Let

(12) $$p=\left(p_{1},p_{2},p_{3}\right)$$

with

(13) $$p_{1}>0,\;p_{2}>0,\;p_{3}>0$$

and

(14) $$p_{1}+p_{2}+p_{3}=1$$

Using coordinates $\left(p_{1},p_{2}\right)$, the third component is

(15) $$p_{3}=1-p_{1}-p_{2}$$

The Fisher metric on the simplex is

(16) $$g_{ab}=\frac{\delta_{ab}}{p_{a}}+\frac{1}{p_{3}},\;a,b\in \{1,2\}$$

Here, $\delta_{ab}$ is the Kronecker delta. The metric is defined on the interior of the simplex, where all probabilities are strictly positive.

The square-root embedding is

(17) $$p\mapsto \left(2\sqrt{p_{1}},2\sqrt{p_{2}},2\sqrt{p_{3}}\right)$$

This map embeds the probability simplex into the positive octant of a sphere of radius 2. Therefore, the categorical Fisher manifold is locally spherical and has constant positive scalar curvature:

(18) $$R_{simplex}=\frac{1}{2}$$

For a multinomial model with *n* sampled elements, the Fisher metric is scaled by *n*:

(19) $$g_{multinomial}=ng_{categorical}$$

Since scalar curvature rescales inversely under constant rescaling of the metric, the scalar curvature becomes

(20) $$R_{multinomial}=\frac{1}{2n}$$

Thus, the curvature magnitude depends on the effective sample size, but the positive curvature sign is preserved.

In the clinical interpretation used here, the simplex template represents normalized finite-capacity organization. It applies when the observables are fractions or proportions constrained by a fixed total, such as immune-cell fractions, tumor-compartment proportions, organ-burden distributions, or trajectory-motif fractions.

These two templates provide sufficient conditions, not universal laws. A scale-related clinical observable does not automatically imply negative Fisher curvature, and a normalized clinical representation does not automatically imply clinically meaningful positive curvature. Curvature sign becomes interpretable only after the observable family, statistical model, coordinate system, and normalization constraints have been specified.

### 2.3. Relational Embedding of Clinical Observables

To connect the Fisher templates with clinical data structures, patient-state organization is represented through a weighted relational graph

(21) $$G=\left(V,E,C\right)$$

Here, *V* is the set of clinical nodes, *E* is the set of relational edges, and *C* assigns a positive constraint weight to each edge.

The nodes may represent physiological variables, laboratory markers, organ systems, imaging regions, radiomic features, molecular markers, immune components, or temporal trajectory states. The edges may represent statistical dependence, physiological coupling, transition probability, co-variation, treatment-related interaction, or constraint propagation. The weight *C*(*e*) is interpreted as a constraint cost, instability load, resistance, or relational burden associated with edge *e*.

This construction adapts the relational–informational framework previously proposed for emergent geometry and effective spacetime to clinical statistical states [11]. In the present setting, no physical spacetime curvature is assumed. The graph is used only as a representation of clinical-relational organization. Geometry appears after coarse-graining, when graph observables are mapped to statistical families.

For a given graph *G*, define a vector of coarse-grained observables:

(22) $$O\left(G\right)=\left(O_{1}\left(G\right),O_{2}\left(G\right),\ldots ,O_{m}\left(G\right)\right)$$

Examples include mean edge constraint, variance of edge constraints, motif fractions, graph entropy, accessibility cost, local heterogeneity, or compartment proportions. These observables define macroscopic clinical states.

For an exponential-family ensemble written in natural coordinates, one may write

(23) $$P\left(G|\eta \right)=\frac{1}{Z(\eta )}\exp\left[\sum_{i=1}^{m}\eta_{i}O_{i}(G)\right]$$

where $O_{i}(G)$ are coarse clinical-relational observables, $\eta_{i}$ are natural parameters, and Z(η) is the partition function.

Equivalently,

(24) $$P\left(G|\eta \right)=\exp\left[\sum_{i=1}^{m}\eta_{i}O_{i}(G)-\psi (\eta )\right]$$

with

(25) $$\psi \left(\eta \right)=\log Z(\eta )$$

In a constraint-cost formulation, one may instead write

(26) $$P_{\beta ,\lambda }\left(G\right)=\frac{1}{Z(\beta ,\lambda )}\exp\left[-\beta S_{\lambda }(G)\right]$$

where $S_{\lambda }(G)$ is a graph-level clinical action or constraint burden, β controls the strength of the constraint penalty, and λ controls the degree of heterogeneity or instability in the edge-weight structure.

A minimal additive graph action can be written as

(27) $$S_{\lambda }\left(G\right)=\sum_{e\in E}\Phi_{\lambda }\left(C(e)\right)$$

where $\Phi_{\lambda }$ is a monotonic transformation of the edge constraint cost.

In the fluctuation-dominated setting, the relevant graph observables are the mean and variance of local constraint costs. These define an effective location–scale family and are compared with the Gaussian Fisher template. In the normalized finite-capacity setting, the relevant observables are motif fractions or compartment proportions satisfying a simplex constraint. These are compared with the categorical/simplex Fisher template.

### 2.4. Exact Fisher Metrics and Covariance-Based Diagnostic Proxies

A central distinction in this study is the difference between exact Fisher metrics and covariance-based diagnostic proxies.

For an exponential-family model written in natural coordinates *η*, the Fisher metric is

(28) $$g_{ij}^{\left(\eta \right)}=\frac{\partial^{2}\psi }{\partial \eta_{i}\partial \eta_{j}}$$

Equivalently, using standard exponential-family identities, this metric is the covariance matrix of the sufficient statistics,

(29) $$g_{ij}^{\left(\eta \right)}={Cov}_{\eta }\left[O_{i}\left(G\right),O_{j}\left(G\right)\right]$$

Therefore, in natural coordinates, the covariance matrix of sufficient statistics is the Fisher information metric.

However, clinical simulations and graph-based numerical experiments are often parameterized by external controls rather than by natural parameters. Examples include β, λ, graph density, rewiring probability, motif thresholds, coarse-graining scale, and measurement noise level.

Let these external controls be denoted by

(30) $$\alpha =\left(\alpha_{1},\alpha_{2},\ldots ,\alpha_{r}\right)$$

If the natural parameters *η* are smooth functions of α, then the Fisher metric in α-coordinates is obtained by pullback:

(31) $$G_{ab}\left(\alpha \right)=\sum_{i=1}^{m}\sum_{j=1}^{m}\frac{\partial \eta_{i}}{\partial \alpha_{a}}g_{ij}^{\left(\eta \right)}\frac{\partial \eta_{j}}{\partial \alpha_{b}}$$

Thus, empirical covariance matrices can be interpreted as Fisher metrics only when the coordinates are natural parameters or when the pullback from external controls to natural coordinates is explicitly known.

When this map is not available, the empirical covariance matrix is used only as a Fisher-consistent diagnostic proxy. For *M* sampled graph configurations,

(32) $$G_{1},G_{2},\ldots ,G_{M}$$

the sample mean of observable $O_{i}$ is

(33) $${\bar{O}}_{i}=\frac{1}{M}\sum_{m=1}^{M}O_{i}(G_{m})$$

The empirical covariance matrix is then

(34) $$\Sigma_{ij}=\frac{1}{M-1}\sum_{m=1}^{M}\left[O_{i}\left(G_{m}\right)-{\bar{O}}_{i}\right]\left[O_{j}\left(G_{m}\right)-{\bar{O}}_{j}\right]$$

This matrix is used to evaluate local numerical behavior through diagnostic quantities such as the determinant,

(35) det(Σ)

and the condition number,

(36) $$cond\left(\Sigma \right)=\frac{\lambda_{max}(\Sigma )}{\lambda_{min}(\Sigma )}$$

Here, $\lambda_{max}(\Sigma )$ and $\lambda_{min}(\Sigma )$ are the largest and smallest eigenvalues of Σ, respectively.

When numerical regularization is required, a regularized covariance matrix is defined as

(37) $$\Sigma_{\varepsilon }=\Sigma +\varepsilon \frac{tr(\Sigma )}{d}I_{d}$$

where *d* is the dimension of the observable vector, $I_{d}$ is the *d*-dimensional identity matrix, and ε > 0 is a small regularization parameter.

These quantities are useful for detecting non-degenerate regions, near-singular regimes, finite-sample instability, and simplex-induced covariance constraints. They are not presented as exact scalar-curvature reconstructions unless the corresponding Fisher metric and coordinate map are explicitly specified.

When a smooth two-dimensional metric field is available on a parameter grid, a curvature-sign proxy may be computed numerically from finite differences of the metric components. This procedure estimates first and second metric derivatives, constructs Christoffel symbols, computes the Ricci tensor, and contracts it with the inverse metric. In the present graph-based implementation, such quantities are interpreted qualitatively as curvature-sign diagnostics. Grid cells with small determinant, high condition number, or unstable finite-difference behavior are excluded from curvature-sign interpretation.

### 2.5. Riemannian–Laplace Approximation for Tractability

Exact scalar-curvature computation becomes difficult when the statistical family is high-dimensional, non-Gaussian, graph-defined, or available only through simulation. In such settings, a local approximation around a dominant macrostate can provide a tractable geometric representation.

Let L(θ) denote a negative log-likelihood, negative log-posterior, or effective clinical loss function defined on a statistical manifold with metric g(θ). Let $\hat{\theta }$ be a dominant macrostate, defined by

(38) $$\hat{\theta }=\arg\min_{\theta }L(\theta )$$

In Euclidean Laplace approximation, the local behavior of *L* around $\hat{\theta }$ is approximated using the Hessian matrix. On a Riemannian statistical manifold, the analogous construction uses the covariant Hessian,

(39) $$H_{ij}^{R}\left(\hat{\theta }\right)=\nabla_{i}\nabla_{j}L(\theta )\left|\begin{array}{c} \theta =\hat{\theta } \end{array}\right.$$

where ∇ denotes the Levi-Civita connection associated with the metric *g*.

A local approximation is then constructed in the tangent space at $\hat{\theta }$,

(40) $$q(v)\propto \exp\left[-\frac{1}{2}v^{T}H^{R}\left(\hat{\theta }\right)v\right],\;v\in T_{\hat{\theta }}M$$

Points on the manifold are recovered locally through the exponential map,

(41) $$\theta \approx {Exp}_{\hat{\theta }}(v)$$

In the present framework, the Riemannian–Laplace approximation has three roles. First, it provides a practical approximation for high-dimensional clinical statistical manifolds where closed-form curvature expressions are unavailable. Second, it preserves the geometric role of the Fisher metric when local uncertainty is approximated around a dominant macrostate. Third, it clarifies that finite numerical implementations should not be interpreted as exact global curvature reconstructions unless the metric, coordinate system, and approximation domain are explicitly controlled [12,13].

### 2.6. Reproducible Computational Protocol

The computational component follows a fixed reporting protocol because graph-based curvature diagnostics depend on graph size, sampling density, parameter-grid resolution, regularization, and metric conditioning.

For each worked example, the following elements are reported in the corresponding case-study subsection: number of nodes *N*, mean graph degree *k*, graph initialization method, edge-weight distribution, definition of the graph action $S_{\lambda }\left(G\right)$, range of β, range of λ, motif thresholds τ_1_ and τ_2_, number of Monte Carlo steps, burn-in period, sampling interval, number of retained samples, number of independent random seeds, convergence criterion, regularization parameter ε, and software environment.

The generic computational workflow is as follows. First, an admissible graph *G* is initialized with fixed node number *N* and prescribed connectivity constraints. In the illustrative graph implementation, edge directionality may be suppressed to simplify numerical sampling. This simplification is reported explicitly and is not used to make claims about directed clinical causality.

Second, edge weights are initialized from a positive distribution and transformed, when needed, into constraint costs. The transformation is chosen so that larger constraint values correspond to larger instability, resistance, burden, or relational cost.

Third, graph configurations are sampled by local rewrite moves. A rewrite move may update an edge weight, rewire a local connection while preserving graph admissibility, or change motif membership according to predefined thresholds. Proposed moves are accepted or rejected using a Metropolis–Hastings rule based on the graph action $S_{\lambda }\left(G\right)$.

Fourth, after burn-in, graph observables are sampled at fixed intervals. For fluctuation-dominated examples, the retained observables include the mean and variance of local constraint costs. For normalized finite-capacity examples, the retained observables include motif fractions satisfying a simplex constraint.

Fifth, empirical covariance matrices are computed from the sampled observables. These matrices are used to report determinant, condition number, eigenvalue range, and regularized diagnostic stability.

Sixth, when a two-dimensional control grid is used, metric-diagnostic maps are computed across the grid. Curvature-sign proxies are reported only for grid cells that satisfy predefined numerical stability conditions. These include positive determinant after regularization, condition number below a specified threshold, and stable finite-difference behavior across neighboring grid cells.

Seventh, sensitivity analysis is performed by varying graph size *N*, sample size *M*, *β* range, *λ* range, motif thresholds, and regularization *ε*. Results are summarized across independent random seeds as means ± standard deviations. For curvature-sign proxies, the percentage of grid cells with stable negative, stable positive, or unstable or undefined signs is reported.

This protocol separates three levels of evidence. The first level consists of exact analytic Fisher-geometric results for the Gaussian and simplex templates. The second level consists of covariance-based diagnostic proxies in finite graph simulations. The third level consists of clinical interpretation, which remains hypothesis-generating until tested on disease-specific datasets.

## 3. Worked Examples and Case-Study Constructions

### 3.1. Scope of the Worked Examples

The purpose of this section is to show how the curvature templates introduced above can be connected to concrete clinical data structures. The examples are methodological rather than diagnostic. They are designed to illustrate how candidate clinical observables can be mapped to fluctuation-dominated, normalized finite-capacity, and graph-based relational settings.

Three examples are considered. The first uses a fluctuation-dominated observable and maps it to the Gaussian location–scale template. The second uses normalized motif or compartment fractions and maps them to the categorical/simplex template. The third uses a weighted clinical-relational graph and shows how covariance-based diagnostic proxies can be computed when exact Fisher reconstruction is not available.

These examples should be interpreted as operational templates. They do not establish clinical validity, diagnostic performance, or prognostic superiority. Their role is to define sufficient statistical conditions under which Fisher-geometric curvature analysis may be meaningfully applied to clinical observables.

### 3.2. Case Study 1: Fluctuation-Dominated Clinical Observable

Consider a clinical observable measured repeatedly within a patient, across a short trajectory, or across comparable patient states. The observable may represent oxygenation variability, acid–base fluctuation, inflammatory-marker dispersion, radiomic heterogeneity, or another instability-related quantity.

Let the observed values be

(42) $$x_{1},x_{2},\ldots ,x_{T}$$

where *T* is the number of observations.

The empirical mean is

(43) $$\hat{\mu }=\frac{1}{T}\sum_{t=1}^{T}x_{t}$$

The empirical variance is

(44) $${\hat{\sigma }}^{2}=\frac{1}{T-1}\sum_{t=1}^{T}{\left(x_{t}-\hat{\mu }\right)}^{2}$$

The corresponding empirical scale is

(45) $$\hat{\sigma }=\sqrt{{\hat{\sigma }}^{2}}$$

The estimated fluctuation-dominated macrostate is then represented by the two-dimensional microstate

(46) $${\hat{\theta }}_{F}=\left(\hat{\mu },\hat{\sigma }\right)$$

Here, the subscript *F* denotes the fluctuation-dominated template.

If the observable is approximated locally by a Gaussian location–scale family, then

(47) $$X\sim N\left(\mu ,\sigma^{2}\right)$$

Equivalently, the probability density is

(48) $$p\left(x|\mu ,\sigma \right)=\frac{1}{\sqrt{2\pi }\sigma }\exp\left[-\frac{{\left(x-\mu \right)}^{2}}{2\sigma^{2}}\right],\;\sigma >0$$

The Fisher metric of this family is

(49) $$ds^{2}=\frac{1}{\sigma^{2}}d\mu^{2}+\frac{2}{\sigma^{2}}d\sigma^{2}$$

The scalar curvature is

(50) $$R_{F}=-1$$

In this example, the negative curvature does not indicate clinical deterioration by itself. It indicates that the selected observable has been represented by a statistical family with an unconstrained scale direction. Clinical interpretation becomes possible only if the empirical scale $\hat{\sigma }$ has a meaningful relationship to instability, heterogeneity, or fluctuation burden in the specific dataset.

A practical instability index can be defined from the empirical scale as

(51) $${\hat{I}}_{F}=\log\left(1+\hat{\sigma }\right)$$

This index is not a curvature measure. It is a simple observable-level summary that increases with fluctuation amplitude. It is useful only for reporting the magnitude of the empirical fluctuation scale associated with a given patient state.

For a cohort of patients indexed by q=1,…,Q, each patient has a macrostate

(52) $${\hat{\theta }}_{F}^{\left(q\right)}=\left({\hat{\mu }}^{\left(q\right)},{\hat{\sigma }}^{\left(q\right)}\right)$$

The corresponding set of patient macrostates is

(53) $$M_{F}^{emp}=\left\{{\hat{\theta }}_{F}^{\left(q\right)}:q=1,\ldots ,Q\right\}$$

This empirical cloud can be visualized in the $\left(\mu ,\sigma \right)$ plane and interpreted relative to the Gaussian Fisher template. Regions with larger $\hat{\sigma }$ correspond to stronger fluctuation amplitude. However, the analytic curvature remains a property of the Gaussian statistical model, not of the finite empirical scatterplot itself.

In a real clinical application, this case-study construction would require reporting the clinical variable used, the sampling window, the measurement frequency, missing-data handling, preprocessing, and the clinical endpoint against which the fluctuation scale is eventually tested.

### 3.3. Case Study 2: Normalized Finite-Capacity Clinical Observable

The second example considers clinical observables that are naturally expressed as fractions or proportions constrained by a fixed total. Examples include immune-cell fractions, tumor-compartment proportions, radiomic phenotype fractions, organ-burden distributions, or fractions of time spent in different trajectory motifs.

Assume that each patient state is represented by three non-negative components,

(54) $$n_{1},n_{2},n_{3}$$

where the components may represent three clinical compartments, motif classes, or categorical state types.

The total count is

(55) $$n=n_{1}+n_{2}+n_{3}$$

The empirical normalized fractions are

(56) $${\hat{p}}_{i}=\frac{n_{i}}{n},\;i=1,2,3$$

They satisfy

(57) $${\hat{p}}_{1}+{\hat{p}}_{2}+{\hat{p}}_{3}=1$$

with

(58) $${\hat{p}}_{i}>0,\;i=1,2,3$$

The estimated normalized finite-capacity macrostate is therefore

(59) $${\hat{\theta }}_{C}=\left({\hat{p}}_{1},{\hat{p}}_{2},{\hat{p}}_{3}\right)$$

where the subscript *C* denotes the compositional or constrained template.

Because of the normalization constraint, only two coordinates are independent. A convenient coordinate choice is

(60) $${\hat{\theta }}_{C}=\left({\hat{p}}_{1},{\hat{p}}_{2}\right)$$

with

(61) $${\hat{p}}_{3}=1-{\hat{p}}_{1}-{\hat{p}}_{2}$$

For the associated Fisher-geometric template, we use generic simplex coordinates p_1_, p_2_, and p_3_. In empirical applications, the metric is evaluated at $p_{i}={\hat{p}}_{i}$.The Fisher metric on the three-state probability simplex is

(62) $$g_{ab}(p)=\frac{\delta_{ab}}{p_{a}}+\frac{1}{p_{3}},\;a,b\in \{1,2\}$$

The square-root embedding is

(63) $$p\mapsto \left(2\sqrt{p_{1}},2\sqrt{p_{2}},2\sqrt{p_{3}}\right)$$

This embedding maps the simplex to the positive octant of a sphere of radius 2. The corresponding scalar curvature is

(64) $$R_{C}=\frac{1}{2}$$

For a multinomial sample of size *n*, the Fisher metric is rescaled by *n*, and the scalar curvature becomes

(65) $$R_{C}^{\left(n\right)}=\frac{1}{2n}$$

This case study illustrates a different form of clinical organization from Case Study 1. Here, the relevant structure is not an unconstrained fluctuation scale, but a redistribution of finite capacity among competing components. A change in one component necessarily affects at least one other component because the total sum is fixed.

A simple empirical finite-capacity imbalance index can be defined as

(66) $${\hat{I}}_{C}=\sum_{i=1}^{3}{\left({\hat{p}}_{i}-\frac{1}{3}\right)}^{2}$$

This index measures deviation from the uniform composition. It is not a curvature measure, but it provides a simple descriptive summary of how concentrated or imbalanced the finite-capacity state is.

For a cohort of patients, the empirical set of compositional macrostates is

(67) $$M_{C}^{emp}=\left\{{\hat{\theta }}_{C}^{\left(q\right)}:\;q=1,\ldots ,Q\right\}$$

These states lie inside the probability simplex. The Fisher geometry of the simplex provides the appropriate statistical geometry for comparing nearby compositional states. Euclidean distances between raw fractions may be misleading because they ignore the normalization constraint.

As in the fluctuation-dominated case, clinical interpretation requires explicit observable definition. For example, if *p*_1_, *p*_2_, and *p*_3_ represent tumor compartments, then segmentation rules must be specified. If they represent immune-cell fractions, then laboratory or cytometry definitions must be provided. If they represent trajectory motifs, then the motif-classification rule must be stated before curvature interpretation is attempted.

### 3.4. Case Study 3: Graph-Based Relational Clinical Embedding

The third example connects the two curvature templates with a graph-based clinical representation. This example is designed to make explicit how nodes, edges, weights, graph observables, and covariance-based diagnostic proxies can be defined.

A generic clinical graph configuration is represented as

(68) $$G=\left(V,E,C\right)$$

where *V* is the set of clinical nodes, *E* is the set of edges, and *C*(*e*) > 0 is the constraint weight assigned to edge *e*.

For an empirical patient *q*, the reconstructed graph is denoted by

(69) $${\hat{G}}^{\left(q\right)}=\left(V^{\left(q\right)},E^{\left(q\right)},{\hat{C}}^{\left(q\right)}\right)$$

Here, ${\hat{C}}^{\left(q\right)}(e)$ denotes the estimated constraint weight of edge *e* in the patient-specific graph. If a cohort-level graph is used instead of patient-specific graphs, the same notation applies to the reconstructed cohort graph.

As a concrete example, in an intensive-care trajectory application, nodes could represent pH, PaCO_2_, PaO_2_, HCO_3_^−^, SpO_2_, lactate, and respiratory-support state. Edges could be estimated from regularized correlation, mutual information, or transition dependence across predefined time windows. The resulting edge weights could then be normalized into positive clinical-relational constraint costs before computing local constraint burden, graph-derived fluctuation macrostates, and motif fractions. This example is illustrative only; in a real clinical application, node selection, edge-weight estimation, normalization, uncertainty handling, and endpoint definition would have to be pre-specified.

The number of nodes in a generic graph is

(70) $$N=\left|V\right|$$

The number of edges is

(71) $$M_{E}=\left|E\right|$$

For an undirected graph, the mean graph degree is

(72) $$k=\frac{2M_{E}}{N}$$

Each edge has a positive constraint weight,

(73) $$C\left(e\right)>0,\;e\in E$$

A local node-level constraint burden is defined as

(74) $$c\left(v\right)=\frac{1}{d(v)}\sum_{e\sim v}C(e)$$

where d(v) is the degree of node *v* and *e*∼*v* means that edge *e* is incident to node *v*. This definition assumes that *d*(*v*) > 0. Isolated nodes should either be removed before graph construction or handled by a pre-specified rule.

For an empirical patient-specific graph, the corresponding estimated local constraint burden is

(75) $${\hat{c}}^{\left(q\right)}\left(v\right)=\frac{1}{d^{\left(q\right)}(v)}\sum_{e\sim v}{\hat{C}}^{\left(q\right)}(e)$$

The graph-level mean constraint is

(76) $$O_{1}\left(G\right)=\frac{1}{N}\sum_{v\in V}c(v)$$

For patient *q*, the estimated graph-level mean constraint is

(77) $${\hat{O}}_{1}^{\left(q\right)}=O_{1}\left({\hat{G}}^{\left(q\right)}\right)$$

The graph-level constraint variance is

(78) $$O_{2}\left(G\right)=\frac{1}{N-1}\sum_{v\in V}{\left[c\left(v\right)-O_{1}(G)\right]}^{2}$$

For patient *q*, the estimated graph-level constraint variance is

(79) $${\hat{O}}_{2}^{\left(q\right)}=O_{2}\left({\hat{G}}^{\left(q\right)}\right)$$

The graph-derived fluctuation macrostate is

(80) $$O_{F}\left(G\right)=\left(O_{1}\left(G\right),\sqrt{O_{2}(G)}\right)$$

For an empirical patient-specific graph, this becomes

(81) $${\hat{\theta }}_{G,F}^{\left(q\right)}=\left({\hat{O}}_{1}^{\left(q\right)},\sqrt{{\hat{O}}_{2}^{\left(q\right)}}\right)$$

This graph-derived macrostate can be compared with the Gaussian location–scale template, where $O_{1}\left(G\right)$ plays the role of a location-like quantity and $\sqrt{O_{2}(G)}$ plays the role of a scale-like quantity.

To define a finite-capacity graph macrostate, edges are assigned to three motif classes according to two thresholds,

(82) $$\tau_{1}<\tau_{2}$$

The motif class of an edge is defined by

(83) $$m\left(e\right)=\left\{\begin{array}{c} 1,\;C\left(e\right)<\tau_{1},\\ 2,\;\tau_{1}\leq C\left(e\right)<\tau_{2}\\ 3,\;C\left(e\right)\geq \tau_{2}. \end{array}\right.,$$

The number of edges in each motif class is

(84) $$n_{i}\left(G\right)=\left|\left\{e\in E:\;m\left(e\right)=i\right\}\right|,\;i=1,2,3$$

The corresponding motif fractions are

(85) $$p_{i}\left(G\right)=\frac{n_{i}(G)}{M_{E}},\;i=1,2,3$$

They satisfy

(86) $$p_{1}\left(G\right)+p_{2}\left(G\right)+p_{3}\left(G\right)=1$$

The graph-derived compositional macrostate is

(87) $$O_{C}\left(G\right)=\left(p_{1}\left(G\right),p_{2}\left(G\right),p_{3}\left(G\right)\right)$$

For an empirical patient-specific graph, the estimated graph-derived compositional macrostate is

(88) $${\hat{\theta }}_{G,C}^{\left(q\right)}=\left({\hat{p}}_{1}^{\left(q\right)},{\hat{p}}_{2}^{\left(q\right)},{\hat{p}}_{3}^{\left(q\right)}\right)$$

where

(89) $${\hat{p}}_{i}^{\left(q\right)}=p_{i}\left({\hat{G}}^{\left(q\right)}\right),\;i=1,2,3$$

This graph-derived compositional macrostate can be compared with the categorical/simplex template. The quantities $p_{i}(G)$ are exact functions of a graph configuration, whereas ${\hat{p}}_{i}^{\left(q\right)}$ are empirical estimates obtained from the reconstructed graph of patient *q*.

Thus, the graph-based construction produces two types of macrostates. The first,

(90) $${\hat{\theta }}_{G,F}^{\left(q\right)}=\left({\hat{O}}_{1}^{\left(q\right)},\sqrt{{\hat{O}}_{2}^{\left(q\right)}}\right)$$

is a fluctuation-type graph macrostate. The second,

(91) $${\hat{\theta }}_{G,C}^{\left(q\right)}=\left({\hat{p}}_{1}^{\left(q\right)},{\hat{p}}_{2}^{\left(q\right)},{\hat{p}}_{3}^{\left(q\right)}\right)$$

is a normalized finite-capacity graph macrostate.

The first can be interpreted relative to the Gaussian location–scale Fisher template, while the second can be interpreted relative to the categorical/simplex Fisher template. In both cases, the graph-derived quantities are empirical or simulated observables; they are not themselves exact Fisher metrics unless the corresponding statistical model and coordinate map are explicitly specified.

### 3.5. Sampling of Graph Configurations

To generate an ensemble of graph configurations, a graph-level constraint action is defined as

(92) $$S_{\lambda }\left(G\right)=\sum_{e\in E}\Phi_{\lambda }(C\left(e\right))$$

In the illustrative implementation, a simple heterogeneity-sensitive transformation is

(93) $$\Phi_{\lambda }\left(C\left(e\right)\right)=C\left(e\right)+\lambda {\left[C\left(e\right)-\bar{C}\right]}^{2}$$

where λ≥0 controls the penalty associated with edge-weight heterogeneity, and

(94) $$\bar{C}=\frac{1}{M_{E}}\sum_{e\in E}C(e)$$

is the mean edge constraint.

The probability of a graph configuration is

(95) $$P_{\beta ,\lambda }\left(G\right)=\frac{1}{Z(\beta ,\lambda )}\exp\left[-\beta S_{\lambda }\left(G\right)\right]$$

where β>0 controls the strength of the constraint penalty and Z(β,λ) is the normalizing partition function.

Graph configurations are sampled using local proposals. If *G* is the current graph and *G’* is a proposed graph, the Metropolis acceptance probability is

(96) $$A\left(G\to G^{\prime }\right)=\min\left\{1,\exp\left[-\beta (S_{\lambda }\left(G^{\prime }\right)-S_{\lambda }\left(G\right))\right]\right\}$$

For symmetric local proposals, this acceptance rule satisfies detailed balance with respect to $P_{\beta ,\lambda }\left(G\right)$. For non-symmetric or state-dependent proposal kernels, the corresponding Metropolis–Hastings proposal ratio must be included.

After burn-in, the retained sampled graphs are denoted by

(97) $$G_{1},G_{2},\ldots ,G_{M}$$

where *M* is the number of retained graph samples.

For a graph observable vector *O*(*G*), the sample mean is

(98) $$\bar{O}=\frac{1}{M}\sum_{m=1}^{M}O(G_{m})$$

The sample covariance matrix of the graph observables is

(99) $$\Sigma_{O}=\frac{1}{M-1}\sum_{m=1}^{M}\left[O\left(G_{m}\right)-\bar{O}\right]{\left[O\left(G_{m}\right)-\bar{O}\right]}^{T}$$

The matrix $\Sigma_{O}$ is interpreted as an exact Fisher metric only if the sampled observables are sufficient statistics expressed in natural coordinates. Otherwise, it is treated as a covariance-based diagnostic proxy.

### 3.6. Diagnostic Quantities Reported for the Worked Examples

For the fluctuation-dominated example, the reported quantities are

(100) $$\hat{\mu },\;\hat{\sigma },\;{\hat{I}}_{F},\;R_{F}=-1$$

Here, $R_{F}=-1$ is the analytic scalar curvature of the Gaussian location–scale Fisher template, not a curvature estimate from finite empirical data.

For the normalized finite-capacity example, the reported quantities are

(101) $${\hat{p}}_{1},\;{\hat{p}}_{2},\;{\hat{p}}_{3},\;{\hat{I}}_{C},\;R_{C}=\frac{1}{2}$$

Here, $R_{C}=\frac{1}{2}$ is the analytic scalar curvature of the categorical/simplex Fisher template.

For a multinomial sample of size *n*, the corresponding curvature scale is reported as

(102) $$R_{C}^{\left(n\right)}=\frac{1}{2n}$$

For the graph-based example, the reported diagnostic quantities include the determinant of the covariance proxy,

(103) $$D_{O}=det(\Sigma_{O})$$

the condition number,

(104) $$\kappa_{O}=\frac{\lambda_{max}(\Sigma_{O})}{\lambda_{min}(\Sigma_{O})}$$

and the regularized covariance proxy,

(105) $$\Sigma_{O,\varepsilon }=\Sigma_{O}+\varepsilon \frac{tr\left(\Sigma_{O}\right)}{d}I_{d}$$

Here, $\lambda_{max}(\Sigma_{O})$ and $\lambda_{min}(\Sigma_{O})$ are the largest and smallest eigenvalues of $\Sigma_{O}$, *d* is the dimension of the observable vector, $I_{d}$ is the *d*-dimensional identity matrix, and ε>0 is a small regularization parameter.

Curvature-sign proxies, when computed, are reported only for parameter-grid regions satisfying the numerical stability conditions

(106) $$D_{O}>0,\;\kappa_{O}<\kappa_{max}$$

together with stable finite-difference behavior across neighboring grid cells.

The distinction between analytic template curvature and numerical diagnostic proxies is maintained throughout the analysis. This distinction prevents the graph-based example from being overinterpreted as an exact reconstruction of Fisher scalar curvature.

### 3.7. Reproducibility Parameters

For reproducibility, each worked example reports the data structure, observable definitions, preprocessing rules, and numerical parameters. For graph-based simulations, the following parameters are specified:

(107) $$N,{\;M}_{E},\;k,\;\beta_{min},\;\beta_{max},{\;\lambda }_{min},{\;\lambda }_{max},\;\tau_{1},\;\tau_{2},\;M,\;\varepsilon$$

Here, *N* is the number of nodes, $M_{E}$ is the number of edges, *k* is the mean degree, β is the constraint-penalty parameter, λ is the heterogeneity-control parameter, $\tau_{1}$ and $\tau_{2}$ are motif thresholds, *M* is the number of retained graph samples, and ε is the covariance regularization parameter.

The reporting table for each simulation includes the initialization method, edge-weight distribution, number of Monte Carlo steps, burn-in period, sampling interval, number of independent random seeds, convergence criterion, and software environment.

This section therefore establishes how the analytical templates and the graph-based diagnostic procedures are operationalized. The next section reports the main analytic and numerical results derived from these constructions.

## 4. Main Results

### 4.1. Analytical Curvature Result for the Fluctuation-Dominated Template

The first analytical result concerns the Gaussian location–scale family used as the fluctuation-dominated curvature template. The Fisher line element of this family is

(108) $$ds^{2}=\frac{1}{\sigma^{2}}d\mu^{2}+\frac{2}{\sigma^{2}}d\sigma^{2}$$

The corresponding scalar curvature is

(109) $$R_{Gaussian}=-1$$

This is an exact Fisher-geometric result for the Gaussian location–scale statistical manifold. In the present framework, it is used as a controlled template for clinical observables whose interpretation depends on both an average level and a fluctuation scale. The negative curvature is therefore not interpreted as a direct marker of clinical deterioration. Rather, it identifies the geometry of a statistical family with an unconstrained scale direction. Clinical interpretation becomes meaningful only when the empirical scale $\hat{\sigma }$ is shown to reflect disease-relevant variability, dispersion, heterogeneity, or instability burden.

For the worked fluctuation-dominated example, the empirical patient state is represented by

(110) $${\hat{\theta }}_{F}=\left(\hat{\mu ,}\hat{\sigma }\right)$$

and the empirical fluctuation index is

(111) $${\hat{I}}_{F}=\log\left(1+\hat{\sigma }\right)$$

The quantity ${\hat{I}}_{F}$ is not a curvature measure. It reports the magnitude of the empirical fluctuation scale associated with a given patient state. The curvature statement remains the analytical statement in Equation (109).

### 4.2. Analytical Curvature Result for the Normalized Finite-Capacity Template

The second analytical result concerns the categorical/simplex family used as the normalized finite-capacity curvature template. For a three-state probability vector,

(112) $$p=\left(p_{1},p_{2},p_{3}\right),\;p_{1}+p_{2}+p_{3}=1$$

the square-root embedding maps the simplex to the positive octant of a sphere of radius 2. The scalar curvature of the categorical Fisher manifold is

(113) $$R_{simplex}=\frac{1}{2}$$

For a multinomial model with *n* sampled elements, the Fisher metric is scaled by *n*, and the scalar curvature becomes

(114) $$R_{multinomial}=\frac{1}{2n}$$

Thus, the positive curvature sign is preserved, while the curvature magnitude depends on the effective sample size. In the present framework, this template applies to observables expressed as fractions, proportions, or motif distributions constrained by a fixed total. Examples include immune-cell fractions, tumor-compartment proportions, radiomic phenotype fractions, organ-burden distributions, or fractions of time spent in trajectory motifs.

For the worked compositional example, the empirical finite-capacity imbalance index is

(115) $${\hat{I}}_{C}=\sum_{i=1}^{3}{\left({\hat{p}}_{i}-\frac{1}{3}\right)}^{2}$$

This index is not a curvature measure. It summarizes how far the empirical composition is from the uniform distribution. The curvature statement remains the analytical statement in Equation (113) (Table 1).

### 4.3. Graph-Derived Macrostates and Numerical Diagnostic Proxies

The graph-based implementation was used to examine whether the proposed fluctuation and compositional observables produce numerically stable covariance-based diagnostic proxies. The baseline graph configuration used

(116) $$N=30,\;M_{E}=60,\;k=4$$

The numerical grid was defined by

(117) β∈{0.2,0.5,1.0,2.0}, λ∈{0.0,0.5,1.0,2.0}

The motif thresholds were

(118) $$\tau_{1}=0.8,\;\tau_{2}=1.2$$

Each grid point was sampled using three independent random seeds. Each run used 3500 Monte Carlo steps, a burn-in period of 1000 steps, a sampling interval of 25, and 100 retained graph samples. The covariance regularization parameter was

(119) $$\varepsilon =10^{-6}$$

The graph-derived fluctuation macrostate was represented by

(120) $$O_{F}\left(G\right)=\left(O_{1}\left(G\right),\sqrt{O_{2}\left(G\right)}\right)$$

where $O_{1}\left(G\right)$ is the mean local constraint burden and $\sqrt{O_{2}\left(G\right)}$ is the scale-like graph fluctuation observable. The graph-derived compositional macrostate was represented by

(121) $$O_{C}\left(G\right)=\left(p_{1}\left(G\right),p_{2}\left(G\right),p_{3}(G)\right)$$

where $p_{1}\left(G\right),p_{2}\left(G\right)$ and $p_{3}(G)$ are the motif fractions induced by the thresholds $\tau_{1}$ and $\tau_{2}$.

**Figure 1:**
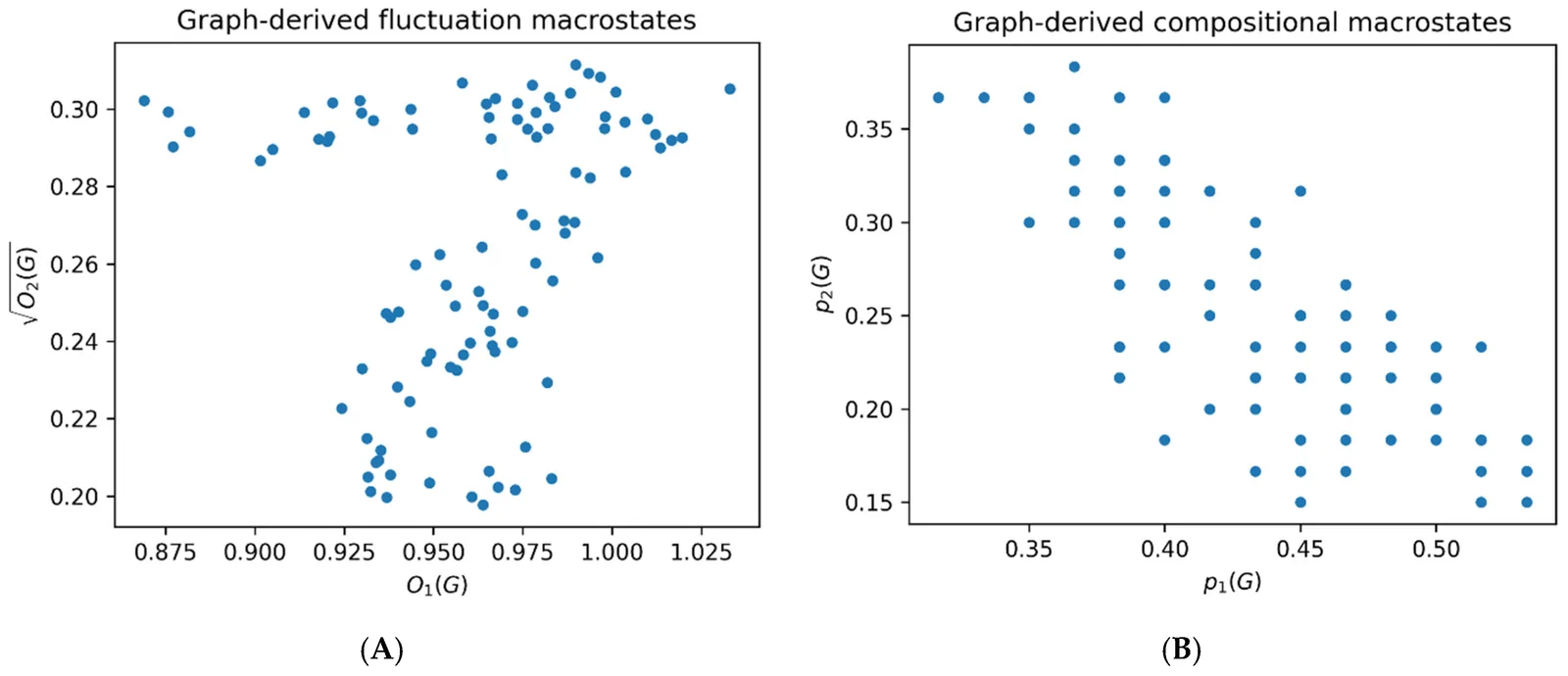
Graph-derived macrostates in the illustrative simulation. (**A**) Fluctuation-type macrostates represented in the $\left(O_{1}\left(G\right),\sqrt{O_{2}\left(G\right)}\right)$ plane. Each point corresponds to one retained graph sample from the illustrative ensemble. (**B**) Compositional macrostates represented in the $\left(p_{1}\left(G\right),p_{2}\left(G\right)\right)$ plane, with $p_{3}(G)=1-p_{1}(G)-p_{2}(G)$ determined by the simplex constraint. The figure illustrates that the graph construction generates both fluctuation-type and normalized finite-capacity state representations.

Representative graph-derived macrostates are shown in Figure 1.

The fluctuation covariance proxy was computed from $\left(O_{1}\left(G\right),\sqrt{O_{2}\left(G\right)}\right)$ and denoted by

(122) $$\Sigma_{F}$$

The compositional covariance proxy was computed from $\left(p_{1}\left(G\right),p_{2}\left(G\right)\right)$ and denoted by

(123) $$\Sigma_{C}$$

The third motif fraction $p_{3}(G)$ was not included as an independent coordinate because

(124) $$p_{1}\left(G\right)+p_{2}\left(G\right)+p_{3}\left(G\right)=1$$

makes the full three-dimensional covariance matrix singular.

Across the full $\left(\beta ,\lambda \right)$ grid, the Metropolis acceptance rate ranged from 0.864 to 0.988, indicating adequate sampling across all tested configurations. The graph-derived mean constraint $O_{1}\left(G\right)$ ranged from approximately 0.608 to 1.208, while the graph-derived fluctuation scale $\sqrt{O_{2}\left(G\right)}$ ranged from approximately 0.164 to 0.363. The motif fractions also varied across the grid: $p_{1}\left(G\right)$ ranged from approximately 0.318 to 0.712, while $p_{3}\left(G\right)$ ranged from approximately 0.058 to 0.459.

The determinant of the fluctuation covariance proxy ranged from

(125) $$7.08\times 10^{-7}to\;5.07\times 10^{-6}$$

and its condition number ranged from

(126) 2.91 to 39.52

The determinant of the compositional covariance proxy ranged from

(127) $$2.10\times 10^{-6}to\;7.48\times 10^{-6}$$

and its condition number ranged from

(128) 3.34 to 47.92

The stability rate was 1.0 for both fluctuation and compositional covariance proxies across all tested grid cells. The complete grid-based numerical diagnostics are summarized in Table 2.

**Figure 2:**
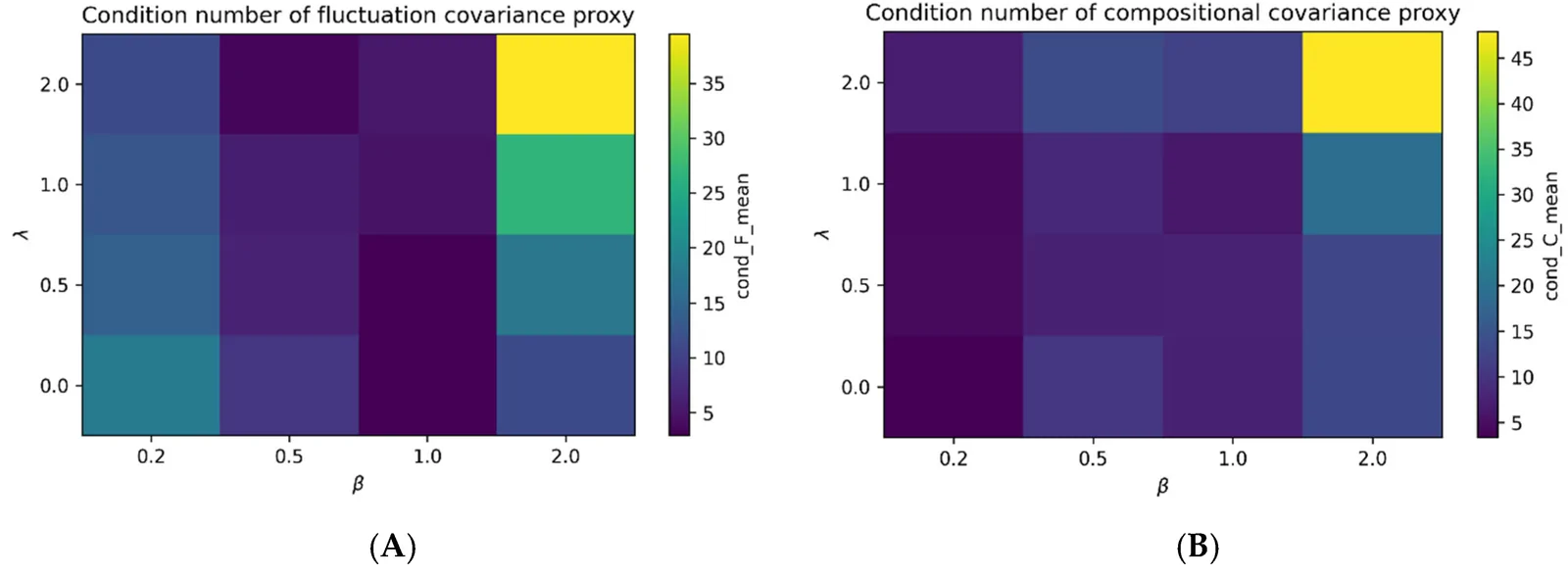
Condition-number maps for graph-based covariance diagnostics. (**A**) Heat map of the condition number $\kappa_{F}=\frac{\lambda_{max}(\Sigma_{F})}{\lambda_{min}(\Sigma_{F})}$ for the fluctuation covariance proxy across the $\left(\beta ,\lambda \right)$ grid. (**B**) Heat map of the condition number $\kappa_{C}=\frac{\lambda_{max}(\Sigma_{C})}{\lambda_{min}(\Sigma_{C})}$ for the compositional covariance proxy across the same grid. Low-to-moderate condition numbers identify parameter regions where the covariance proxy is numerically well conditioned. Higher values identify regimes where finite-sample geometry becomes more sensitive to sampling and regularization.

The condition-number maps are shown in Figure 2. These maps provide a direct visual diagnostic of metric conditioning across the $\left(\beta ,\lambda \right)$ grid.

The combined covariance proxy, computed from $\left(O_{1}\left(G\right),\sqrt{O_{2}\left(G\right)},p_{1}\left(G\right),p_{2}\left(G\right)\right)$, also remained numerically stable under the adopted criterion. Its determinant was smaller, ranging from approximately 4.58 × 10^−13^ to 1.69 × 10^−12^, and its condition number ranged from approximately 25.87 to 323.24. This behavior is to be expected because the combined observable vector mixes fluctuation-type and compositional quantities with different scales and constraint structures. Therefore, the combined covariance proxy is useful as a numerical diagnostic, but it should not be interpreted as an exact Fisher metric.

### 4.4. Sensitivity Analysis

Sensitivity analysis was performed by varying graph size, constraint penalty, heterogeneity penalty, and motif thresholds. The baseline configuration was

(129) $$N=30,\;M_{E}=60,\;k=4,\;\beta =1.0,\;\lambda =0.5,\;\tau_{1}=0.8,\;\tau_{2}=1.2$$

The baseline fluctuation covariance proxy had mean condition number $\kappa_{F}=8.30$, and the baseline compositional covariance proxy had mean condition number $\kappa_{C}=7.20$. The corresponding mean finite-capacity imbalance was ${\hat{I}}_{C}=0.089$.

Increasing the constraint penalty to β=2.0 increased the fluctuation condition number to 47.42 and the compositional condition number to 29.69. It also increased the compositional imbalance to 0.242, indicating that stronger constraint penalization can push the graph ensemble toward more imbalanced motif distributions.

Reducing the constraint penalty to β=0.2 produced a lower compositional imbalance of 0.031, consistent with a more diffuse motif distribution. Increasing the heterogeneity-control parameter to λ=2.0 increased both condition numbers, with $\kappa_{F}=24.92$ and $\kappa_{C}=21.88$, indicating that stronger heterogeneity penalization changes the numerical conditioning of both diagnostic sectors.

Graph size also affected the diagnostic quantities. The smaller graph (N = 20, M_E_ = 40) had $\kappa_{F}=2.89$ and $\kappa_{C}=9.05$, while the larger graph (N = 40, M_E_ = 80) had $\kappa_{F}=5.45$ and $\kappa_{C}=10.16$. In both cases, the covariance proxies remained stable.

Motif-threshold sensitivity was also observed. Wider thresholds ${(\tau }_{1}=0.7,\;\tau_{2}=1.3)$ produced a lower imbalance of 0.059, while narrower thresholds $\left(\tau_{1}=0.9,\;\tau_{2}=1.1\right)$ produced a higher imbalance of 0.146. This confirms that motif-class definitions affect the normalized finite-capacity sector and must be reported explicitly in any clinical application. The complete sensitivity results are summarized in Table 3.

Across all sensitivity configurations, both fluctuation and compositional covariance proxies satisfied the numerical stability criterion. However, the condition numbers and imbalance values changed with β, λ, graph size, and motif thresholds. This indicates that the diagnostic quantities are reproducible under the tested settings, but they remain implementation-dependent numerical summaries rather than intrinsic clinical biomarkers.

### 4.5. Evidence-Level Interpretation of the Results

The results separate three levels of evidence.

First, the curvature values of the Gaussian and categorical/simplex templates are exact analytical Fisher-geometric results. These results establish two controlled curvature templates: a negative-curvature fluctuation-dominated template and a positive-curvature normalized finite-capacity template.

Second, the graph-based simulation provides covariance-based diagnostic proxies. These proxies demonstrate the non-degeneracy, metric conditioning, and sensitivity behavior of the proposed graph-derived observables. They do not reconstruct exact Fisher scalar curvature unless the corresponding natural-coordinate structure or coordinate pullback is explicitly specified.

Third, the clinical interpretation remains hypothesis-generating. The analytical curvature signs and graph-based numerical diagnostics indicate how clinical observables could be organized geometrically, but they do not establish diagnostic accuracy, prognostic value, or superiority over conventional clinical or machine learning models. The three evidence levels are summarized in Table 4.

Overall, the results support the internal consistency of the proposed framework. The analytical templates provide controlled Fisher-geometric curvature signs, while the graph-based implementation shows how clinical-relational observables can be sampled and evaluated through reproducible covariance-based diagnostic proxies. The framework therefore provides a methodological basis for future disease-specific validation, but it does not yet establish curvature-derived quantities as clinical biomarkers.

## 5. Discussion

### 5.1. What Is New and What Is Not New

This study proposes a clinical-relational operationalization of established Fisher-geometric curvature templates. The main contribution is not the derivation of new Fisher manifolds. The Gaussian location–scale family and the categorical/simplex family are known statistical manifolds with established Fisher-geometric properties. Their use here is therefore not presented as a mathematical discovery.

The novelty lies in the way these established geometries are used as controlled templates for clinical state organization. The framework separates three levels that are often conflated in conceptual modeling: exact Fisher-geometric structure, computational diagnostic implementation, and clinical interpretation. The Gaussian location–scale family is used as a negative-curvature template for fluctuation-dominated observables. The categorical/simplex family is used as a positive-curvature template for normalized finite-capacity observables. A graph-based relational embedding is then introduced to show how clinical variables, interactions, constraints, and motif fractions may be mapped into these two template classes.

This separation is central to the revised framework. The analytical curvature values,

$$R_{Gaussian}=-1$$

and

$$R_{simplex}=\frac{1}{2}$$

are exact Fisher-geometric results within their respective statistical models. By contrast, the covariance matrices computed in the graph-based numerical example are not automatically exact Fisher metrics. They are interpreted as covariance-based diagnostic proxies unless the natural-coordinate structure or coordinate pullback is explicitly specified. This distinction prevents overinterpretation of numerical covariance maps as direct scalar-curvature reconstructions.

The work is therefore best understood as a methodological bridge. It does not claim that clinical disease states are literally Gaussian manifolds, spherical simplexes, or physical curved spaces. Rather, it proposes that selected families of clinical observables may be organized through statistical geometries whose curvature signs are analytically controlled. This provides a structured language for distinguishing fluctuation-dominated, finite-capacity, and relationally embedded clinical state representations.

### 5.2. Curvature Templates as Sufficient Conditions, Not Universal Laws

A central point of the framework is that curvature sign is not interpreted as a universal clinical law. A negative Fisher curvature does not automatically imply instability, deterioration, or poor prognosis. Similarly, a positive Fisher curvature does not automatically imply stability, compensation, or a favorable outcome. These interpretations become meaningful only after the observable family, statistical model, coordinate system, and clinical endpoint have been explicitly defined.

The Gaussian location–scale template provides a sufficient condition for negative Fisher curvature in a two-parameter statistical family with a location direction and an unconstrained scale direction. This makes it suitable as a reference geometry for observables whose clinical meaning depends on variability, dispersion, heterogeneity, or fluctuation burden. However, the empirical scale parameter $\hat{\sigma }$ must be clinically meaningful in the specific dataset. If $\hat{\sigma }$ reflects measurement noise, inconsistent sampling, preprocessing artifacts, or unmodeled confounding, then the curvature template remains mathematically valid but clinically uninformative.

The simplex template provides a sufficient condition for positive Fisher curvature in normalized categorical or compositional representations. This makes it suitable for variables constrained by a fixed total, such as immune-cell fractions, tumor-subregion proportions, organ-burden distributions, or trajectory-motif fractions. However, not every normalized vector has a meaningful clinical geometry. The components must correspond to interpretable categories, compartments, or motifs, and the normalization constraint must reflect a clinically relevant finite-capacity structure rather than arbitrary rescaling.

The graph-based example extends this idea by showing how relational observables may be coarse-grained into fluctuation-type and compositional-type macrostates. The graph construction does not create clinical validity by itself. It only defines a reproducible pathway from nodes, edges, weights, and motif thresholds to candidate state-space observables. The value of this construction depends on whether the graph nodes and edges are clinically justified and whether the resulting observables add information beyond conventional predictors.

For these reasons, the curvature templates should be viewed as sufficient mathematical templates for candidate clinical organization, not as universal disease laws. Their purpose is to constrain interpretation, not to replace empirical validation.

### 5.3. Relationship to Machine Learning and Clinical Prediction

The proposed framework is complementary to conventional statistical learning and machine learning approaches. Most clinical prediction models represent patients as points in a high-dimensional feature space and optimize discrimination, calibration, or risk estimation. This approach has produced substantial progress in radiomics, intensive-care modeling, precision medicine, and multimodal prediction. However, such models do not usually ask how the statistical distinguishability of nearby patient states is organized across the state space.

Fisher information geometry adds a different layer of analysis. It focuses on how probability distributions change locally when clinical state parameters vary. In this sense, the framework does not compete with machine learning. It may instead provide additional structure for interpreting learned representations, trajectory embeddings, radiomic phenotypes, or graph-derived patient states.

Related data-analysis approaches have also shown how empirical biological trajectories can be connected to stochastic modeling and prediction. For example, Meyer et al. used data-driven stochastic models to analyze directedness, correlations, daily cycles, and prediction in animal movement trajectories [14]. The present framework is different in scope: it does not aim to predict trajectories directly but to provide a Fisher-geometric layer for organizing candidate clinical state variables according to fluctuation-dominated, compositional, and graph-derived structures.

For example, a machine learning model may identify a set of variables associated with an outcome. The proposed framework asks whether those variables form a fluctuation-dominated family, a finite-capacity compositional family, or a relational graph-derived state. If a clinically meaningful fluctuation scale is present, the Gaussian location–scale template may provide a reference geometry for studying instability. If the variables are fractions constrained by a fixed total, the simplex template may provide a more appropriate geometry than a Euclidean feature space. If the variables are relational, graph-derived covariance diagnostics may help identify non-degenerate regions, unstable parameter regimes, or sensitivity to graph construction.

This geometric layer may also support model audit and feature interpretation. Condition numbers, covariance determinants, simplex constraints, and regularized diagnostic proxies can reveal when a representation is numerically degenerate, highly sensitive to thresholds, or unstable under sampling. These diagnostics are not substitutes for clinical validation metrics such as AUC, calibration, decision-curve analysis, or external validation. Rather, they provide information about the internal organization and numerical stability of candidate clinical state representations.

Thus, the framework should be positioned as an interpretability and hypothesis-generation layer. Its future value depends on whether curvature-derived or geometry-derived quantities improve understanding, robustness, or predictive performance when tested alongside standard clinical and machine learning models.

### 5.4. Limitations

This study has several limitations.

1. The framework is methodological and hypothesis-generating. It is not a validated diagnostic, prognostic, or therapeutic model. The worked examples demonstrate how clinical observables could be mapped to Fisher-geometric templates, but they do not establish disease-specific clinical utility.
2. The analytical curvature results belong to idealized statistical families. The Gaussian location–scale and categorical/simplex manifolds provide controlled reference geometries, but real clinical data may not follow these families exactly. Clinical measurements are often noisy, irregularly sampled, censored, confounded, treatment-dependent, and affected by missingness. Therefore, any future application must test whether the assumed statistical family is appropriate for the clinical observable under study.
3. Covariance-based graph diagnostics are not exact Fisher scalar-curvature reconstructions unless additional conditions are satisfied. In exponential-family models written in natural coordinates, covariance matrices of sufficient statistics correspond to Fisher information. However, graph simulations are often parameterized by external controls such as β, λ, graph density, motif thresholds, or regularization parameters. Without an explicit natural-parameter map or coordinate pullback, the resulting covariance matrices should be interpreted only as Fisher-consistent diagnostic proxies.
4. The graph-based implementation is intentionally simplified. The illustrative simulation uses a controlled graph ensemble, positive edge weights, motif thresholds, and covariance diagnostics. In real clinical systems, graph directionality, temporal order, causal asymmetry, measurement uncertainty, treatment effects, and multimodal data integration may all be important. Suppressing directionality can make the numerical demonstration tractable, but it limits causal and mechanistic interpretation.
5. Motif definitions and thresholds influence the compositional sector. The sensitivity analysis shows that changing $\tau_{1}$ and $\tau_{2}$ changes the imbalance of motif fractions. Therefore, motif thresholds cannot be treated as neutral technical choices. In clinical applications, they must be pre-specified, justified biologically or statistically, and tested for robustness.
6. The numerical outputs depend on simulation settings. Graph size, mean degree, sampling length, burn-in, proposal scale, β, λ, threshold choice, number of retained samples, random seeds, and covariance regularization all influence diagnostic quantities. The reporting protocol introduced here reduces ambiguity, but it does not eliminate implementation dependence.
7. The framework does not yet show incremental predictive value over conventional models. A full clinical validation would require disease-specific datasets, pre-defined endpoints, independent validation cohorts, and comparison with standard statistical or machine learning approaches. Geometry-derived quantities should be tested for discrimination, calibration, decision utility, robustness, and added value beyond established clinical predictors.

These limitations are not incidental. They define the intended status of the framework. The present study provides a controlled mathematical and computational language for clinical state organization, not a finished clinical model.

### 5.5. Future Validation

Future work should move from methodological templates to disease-specific validation. Several application domains are suitable for this transition.

One natural direction is longitudinal intensive-care modeling. Physiological and blood-gas trajectories may contain fluctuation-dominated observables whose empirical scale is clinically meaningful. In such settings, the Gaussian location–scale template could be tested against endpoints such as deterioration, recovery, ventilation requirement, and mortality. The key question would be whether fluctuation-scale geometry adds information beyond conventional trajectory features and severity scores.

A second direction is radiomics and imaging-derived heterogeneity. Tumor regions, radiomic phenotypes, and segmentation-derived compartments may be represented either as fluctuation-dominated features or as normalized finite-capacity compositions. The simplex template may be particularly relevant when tumor subregions are expressed as proportions of a total lesion volume. Future work should test whether a simplex-aware geometry improves interpretation or prediction compared with Euclidean analysis of raw fractions.

A third direction is immunological and molecular profiling. Immune-cell fractions, molecular subtype proportions, and compositional biomarker panels naturally satisfy normalization constraints. These data structures are well suited for testing whether Fisher-simplex geometry provides useful state-space organization and whether finite-capacity imbalance indices correlate with clinical phenotype, treatment response, or survival.

A fourth direction is graph-based multimodal clinical modeling. Clinical graphs may combine laboratory markers, imaging regions, molecular features, treatment variables, and temporal states. In this setting, the proposed graph-derived fluctuation and compositional macrostates could be evaluated as intermediate representations. Future studies should compare different graph-construction rules, including correlation networks, mutual information networks, transition graphs, probabilistic graphical models, and clinically defined relational structures.

Future validation should follow a strict reporting protocol. The clinical variables, preprocessing rules, graph-construction method, edge-weight definition, motif thresholds, statistical family, coordinate system, numerical regularization, and validation endpoint should all be specified before outcome testing. Geometry-derived quantities should then be evaluated for stability, interpretability, and incremental predictive value.

The most important future test is whether the proposed geometric layer changes clinical understanding or improves model performance. If curvature-derived or geometry-derived quantities remain redundant with conventional features, their value will be mainly conceptual. If they identify clinically meaningful instability, constraint, or transition regimes not captured by standard models, then Fisher-geometric clinical state analysis may become a useful complement to existing predictive frameworks.

## 6. Conclusions

This study developed a clinical-relational interpretation of established Fisher-geometric curvature templates and showed how they can be operationalized through worked examples and graph-based diagnostic procedures.

The main conclusions are:

- The Gaussian location–scale family provides an exact negative-curvature Fisher template, with

$$R_{Gaussian}=-1$$

and can be used as a controlled reference geometry for fluctuation-dominated clinical observables.

- The categorical/simplex family provides an exact positive-curvature Fisher template, with

$$R_{simplex}=\frac{1}{2}$$

and can be used as a controlled reference geometry for normalized finite-capacity clinical observables.

- For multinomial compositional data, the scalar curvature rescales as

$$R_{multinomial}=\frac{1}{2n}$$

so the positive curvature sign is preserved while the magnitude depends on effective sample size.

- The proposed clinical interpretation is hypothesis-generating. Negative curvature does not automatically imply clinical deterioration, and positive curvature does not automatically imply clinical stability.
- Graph-derived fluctuation and compositional macrostates provide a reproducible way to connect relational clinical structures with Fisher-geometric templates.
- Covariance-based graph diagnostics should be interpreted as numerical proxies unless the corresponding natural-coordinate Fisher metric or coordinate pullback is explicitly specified.
- Sensitivity analysis shows that graph size, constraint parameters, heterogeneity penalties, motif thresholds, and regularization choices influence numerical diagnostics and must be reported transparently.
- The framework does not replace conventional statistical or machine learning models. It provides an additional geometric layer for organizing, interpreting, and testing candidate clinical state representations.
- Future work must validate the framework on disease-specific datasets with explicit endpoints, reproducible preprocessing, independent testing, and comparison against standard predictive models.

Overall, the proposed framework provides a controlled methodological basis for studying clinical state organization through Fisher information geometry. Its value will depend on future empirical validation showing whether curvature-derived or geometry-derived quantities add clinically meaningful information beyond established statistical and machine learning approaches.

## Figures and Tables

**Table 1:** Analytical curvature templates used in the framework.

Template	Coordinates	Fisher Metric	Scalar Curvature	Clinical Interpretation	Evidence Level
Gaussian location–scale	$\left(\mu ,\;\sigma \right)$	$diag\left(1/\sigma^{2},2/\sigma^{2}\right)$	−1	Fluctuation-dominated organization	Exact analytic Fisher geometry
Categorical/simplex	$\left(p_{1},p_{2}\right),$ $p_{3}=1-p_{1}-p_{2}$	$\delta_{ab}/p_{a}+1/p_{3}$	½	Normalized finite-capacity organization	Exact analytic Fisher geometry
Multinomial simplex	$\left(p_{1},p_{2}\right),$ $p_{3}=1-p_{1}-p_{2}$	n times categorical Fisher metric	1/(2n)	Finite-sample compositional organization	Exact analytic Fisher geometry with sample-size rescaling

**Table 2:** Grid-based numerical diagnostics for the fluctuation and compositional covariance proxies. Determinants are reported in units of 10^−6^. Values are means across three independent random seeds.

β	λ	D_F_	$\kappa_{F}$	D_C_	$\kappa_{C}$	${\hat{I}}_{C}$
0.2	0.0	4.360	17.90	2.108	3.34	0.045
0.2	0.5	3.765	14.14	2.357	4.41	0.041
0.2	1.0	3.746	12.70	2.750	4.23	0.037
0.2	2.0	3.292	11.17	3.085	6.83	0.036
0.5	0.0	2.298	8.68	2.170	10.39	0.035
0.5	0.5	1.593	6.57	2.479	7.38	0.027
0.5	1.0	1.419	5.97	2.402	8.22	0.023
0.5	2.0	0.865	3.49	2.103	13.45	0.017
1.0	0.0	0.708	2.92	2.309	7.51	0.043
1.0	0.5	0.891	2.91	2.425	7.77	0.035
1.0	1.0	1.017	4.81	4.450	6.20	0.028
1.0	2.0	0.972	5.21	5.564	11.90	0.034
2.0	0.0	1.627	11.14	2.813	13.08	0.140
2.0	0.5	2.328	17.39	5.086	12.90	0.157
2.0	1.0	2.696	26.93	6.209	19.53	0.201
2.0	2.0	5.074	39.52	7.478	47.92	0.256

**Table 3:** Sensitivity analysis of graph-based diagnostic quantities. Values are means across three independent random seeds.

Configuration	N	M_E_	β	λ	$\tau_{1}$	$\tau_{2}$	$\kappa_{F}$	$\kappa_{C}$	${\hat{I}}_{C}$
Baseline	30	60	1.0	0.5	0.8	1.2	8.30	7.20	0.089
Smaller graph	20	40	1.0	0.5	0.8	1.2	2.89	9.05	0.141
Larger graph	40	80	1.0	0.5	0.8	1.2	5.45	10.16	0.077
Low (\beta)	30	60	0.2	0.5	0.8	1.2	4.93	9.36	0.031
High (\beta)	30	60	2.0	0.5	0.8	1.2	47.42	29.69	0.242
High (\lambda)	30	60	1.0	2.0	0.8	1.2	24.92	21.88	0.129
Wide thresholds	30	60	1.0	0.5	0.7	1.3	8.30	10.18	0.059
Narrow thresholds	30	60	1.0	0.5	0.9	1.1	8.30	4.68	0.146

**Table 4:** Evidence-level summary of the main results.

Result Block	Quantity Reported	Type of Result	Interpretation	Limitation
Gaussian location–scale template	$R_{Gaussian}=-1$	Exact analytic Fisher curvature	Sufficient template for fluctuation-dominated organization	Does not prove clinical deterioration without dataset-specific validation
Categorical/simplex template	$R_{simplex}=1/2$	Exact analytic Fisher curvature	Sufficient template for normalized finite-capacity organization	Does not imply clinical stability by itself
Multinomial simplex	$R_{multinomial}=1/(2n)$	Exact sample-size rescaling	Curvature sign remains positive; magnitude depends on effective sample size	Requires meaningful definition of (n)
Graph-based fluctuation diagnostics	$det\left(\Sigma_{F}\right),cond\left(\Sigma_{F}\right)$	Covariance-based diagnostic proxy	Numerical stability and non-degeneracy of fluctuation observables	Not exact scalar curvature unless natural-coordinate Fisher metric is known
Graph-based compositional diagnostics	$det\left(\Sigma_{C}\right),cond\left(\Sigma_{C}\right)$	Covariance-based diagnostic proxy	Numerical stability and non-degeneracy of simplex observables	Uses $p_{1}\;and\;p_{2}$ because $p_{1}+p_{2}+p_{3}=1$ makes the full covariance singular
Sensitivity analysis	Variation across $N,\beta ,\lambda ,\tau_{1},\tau_{2}$	Robustness diagnostic	Checks whether diagnostics are stable under numerical choices	Illustrative simulation, not clinical validation

## Data Availability

No real patient-level clinical dataset was analyzed in this methodological study. The illustrative graph-simulation outputs, the Python code used to generate the numerical diagnostics, execution instructions, parameter definitions, and corresponding numerical outputs are available in Supplementary File S1. The analyses were executed in Google Colab using Python 3.12.

## Supplementary Materials

The following supporting information can be downloaded at https://www.mdpi.com/article/10.3390/e28080868/s1, Supplementary File S1: Reproducibility package for the illustrative graph-based numerical diagnostics and sensitivity analyses. The package contains the following files; Code S1. *Supplementary_File_S1_Graph_Diagnostics.py*: Google Colab/Python-compatible script used to generate the graph-based numerical diagnostics, sensitivity analyses, CSV tables, and graphical outputs reported in the manuscript; Text S1. *README_Supplementary_File_S1.txt*: Execution instructions, software dependencies, baseline parameter definitions, output-file descriptions, and limitations of the numerical demonstration; Table S1. *Analytical information-geometric templates* (*table_analytic_templates.csv*). Summary of the Gaussian location–scale, categorical/simplex, and multinomial-simplex models, including their coordinates, Fisher information metrics, scalar curvatures, interpretations, and evidence levels; Table S2. *Seed-level results of the graph-based numerical diagnostics* (*table_grid_results_by_seed.csv*). Results obtained across the complete (\beta)–(\lambda) parameter grid for random seeds 11, 22, and 33, including Monte Carlo acceptance rates, graph observables, covariance determinants, covariance condition numbers, eigenvalue ranges, and numerical-stability indicators; Table S3. *Sampled graph observables across the (\beta)–(\lambda) grid* (*samples_grid_observables.csv*). Retained Monte Carlo samples of the fluctuation observables (O_1), (O_2), and (\sqrt{O_2}), the compositional fractions (p_1), (p_2), and (p_3), and the compositional-imbalance measure, together with the corresponding sampling step, parameter values, and random seed; Table S4. *Aggregated graph-diagnostic results across random seeds* (*table_grid_results_aggregated.csv*). Mean and standard-deviation summaries of the graph observables and covariance-based diagnostic proxies for every combination of the constraint-penalty parameter (\beta) and heterogeneity-control parameter (\lambda); Table S5. *Sensitivity-analysis results* (*table_sensitivity_results.csv*). Effects of changes in graph size, constraint penalty, heterogeneity control, and motif-classification thresholds on the covariance-based fluctuation and compositional diagnostics and on the compositional-imbalance measure; Table S6. *Evidence-level summary of the analytical and numerical results* (*table_evidence_summary.csv*). Classification of each result as an exact analytical Fisher-curvature result, covariance-based numerical diagnostic proxy, or robustness diagnostic, together with its interpretation and principal limitation; Table S7. *Parameters used for the graph-based numerical diagnostics* (*table_reporting_parameters.csv*). Definitions and baseline values of the graph size, number of edges, mean degree, (\beta) and (\lambda) grids, motif thresholds, Monte Carlo settings, random seeds, covariance regularization, and numerical-stability threshold; Figure S1. *Graph-derived fluctuation macrostates* (*figure_graph_fluctuation_macrostates.png*). Scatter plot of the mean local constraint burden (O_1(G)) against the scale-like fluctuation observable (\sqrt{O_2(G)}) for the illustrative configuration (\beta = 1.0), (\lambda = 0.5), and random seed 11. Each point represents one retained Monte Carlo graph sample; Figure S2. *Graph-derived compositional macrostates* (*figure_graph_compositional_macrostates.png*). Scatter plot of the motif fractions (p_1(G)) and (p_2(G)) for the illustrative configuration (\beta = 1.0), (\lambda = 0.5), and random seed 11. The three fractions (p_1), (p_2), and (p_3) classify edge weights below (\tau_1), between (\tau_1) and (\tau_2), and at or above (\tau_2), respectively, with (p_1 + p_2 + p_3 = 1); Figure S3. *Condition number of the fluctuation covariance proxy* (*figure_condition_fluctuation_proxy.png*). Heat map of the mean condition number of the regularized covariance matrix constructed from (O_1(G)) and (\sqrt{O_2(G)}) across the investigated (\beta)–(\lambda) grid. Values are averaged across the three main-grid random seeds; Figure S4. *Condition number of the compositional covariance proxy* (*figure_condition_compositional_proxy.png*). Heat map of the mean condition number of the regularized covariance matrix constructed from (p_1(G)) and (p_2(G)) across the investigated (\beta)–(\lambda) grid. The third fraction is omitted because (p_1 + p_2 + p_3 = 1), which would make the full three-component covariance matrix singular. Values are averaged across the three main-grid random seeds; Figure S5. *Mean compositional imbalance across the parameter grid* (*figure_compositional_imbalance.png*). Heat map of the mean compositional-imbalance measure (\sum_{i=1}^{3}(p_i − 1/3)^2) across the investigated (\beta)–(\lambda) grid, averaged across the three main-grid random seeds. The supplementary numerical outputs are intended to support reproducibility of the methodological demonstration. They represent illustrative covariance-based diagnostic proxies rather than exact Fisher scalar-curvature reconstructions or clinical validation. No real patient-level clinical data are included.

## Appendix A. Gaussian Location–Scale Fisher Geometry

This appendix summarizes the Fisher-geometric derivation for the Gaussian location–scale template used in the main text.

Consider the Gaussian probability density

(A1) $$p\left(x|\mu ,\sigma \right)=\frac{1}{\sqrt{2\pi }\sigma }\exp\left[-\frac{{\left(x-\mu \right)}^{2}}{2\sigma^{2}}\right],\;\sigma >0$$

The log-likelihood for one observation is

(A2) $$l\left(\mu ,\sigma \right)=\log p\left(x|\mu ,\sigma \right)=-\frac{1}{2}\log\left(2\pi \right)-\log\sigma -\frac{{\left(x-\mu \right)}^{2}}{2\sigma^{2}}$$

The score with respect to the location parameter is

(A3) $$\frac{\partial l}{\partial \mu }=\frac{x-\mu }{\sigma^{2}}$$

The score with respect to the scale parameter is

(A4) $$\frac{\partial l}{\partial \sigma }=-\frac{1}{\sigma }+\frac{{\left(x-\mu \right)}^{2}}{\sigma^{3}}$$

Using

(A5) $$E\left[{\left(X-\mu \right)}^{2}\right]=\sigma^{2}$$

and

(A6) $$E\left[{\left(X-\mu \right)}^{4}\right]={3\sigma }^{4}$$

the Fisher metric components are

(A7) $$g_{\mu \mu }=E\left[{\left(\frac{\partial l}{\partial \mu }\right)}^{2}\right]=\frac{1}{\sigma^{2}}$$

(A8) $$g_{\mu \sigma }=E\left[\frac{\partial l}{\partial \mu }\frac{\partial l}{\partial \sigma }\right]=0$$

and

(A9) $$g_{\sigma \sigma }=E\left[{\left(\frac{\partial l}{\partial \sigma }\right)}^{2}\right]=\frac{2}{\sigma^{2}}$$

Therefore, the Fisher line element is

(A10) $$ds^{2}=\frac{1}{\sigma^{2}}d\mu^{2}+\frac{2}{\sigma^{2}}d\sigma^{2}$$

To identify the curvature, introduce the coordinate change

(A11) $$u=\mu ,\;v=\sqrt{2}\sigma$$

Since

(A12) $$\sigma =\frac{v}{\sqrt{2}},\;d\sigma =\frac{dv}{\sqrt{2}}$$

the line element becomes

(A13) $$ds^{2}=2\frac{du^{2}+dv^{2}}{v^{2}}$$

The metric

(A14) $$\frac{du^{2}+dv^{2}}{v^{2}}$$

is the standard Poincaré half-plane metric with Gaussian curvature

(A15) K=−1

Multiplication of the metric by the constant factor 2 rescales Gaussian curvature by 1/2, giving

(A16) $$K_{Gaussian}=-\frac{1}{2}$$

In two dimensions, scalar curvature satisfies

(A17) R=2K

Thus, the scalar curvature of the Gaussian location–scale Fisher manifold is

(A18) $$R_{Gaussian}=-1$$

This result is exact for the Gaussian location–scale statistical manifold. In the clinical framework of the main text, it is used as a controlled negative-curvature template for fluctuation-dominated observables. The derivation itself does not imply any clinical deterioration or prognosis.

## Appendix B. Categorical/Simplex Fisher Geometry

This appendix summarizes the Fisher-geometric derivation for the categorical/simplex template used in the main text.

Consider a three-state categorical distribution

(A19) $$p=\left(p_{1},p_{2},p_{3}\right)$$

with

(A20) $$p_{i}>0,\;i=1,2,3,$$

and

(A21) $$p_{1}+p_{2}+p_{3}=1$$

Using independent coordinates

(A22) $$\theta^{1}=p_{1},\;\theta^{2}=p_{2}$$

the third probability is

(A23) $$p_{3}=1-p_{1}-p_{2}$$

The Fisher metric for a categorical distribution can be written as

(A24) $$g_{ab}=\sum_{i=1}^{3}\frac{1}{p_{i}}\frac{\partial p_{i}}{\partial \theta^{a}}\frac{\partial p_{i}}{\partial \theta^{b}},\;a,b\in \{1,2\}$$

The derivatives are

(A25) $$\frac{\partial p_{1}}{\partial p_{1}}=1,\;\frac{\partial p_{1}}{\partial p_{2}}=0,$$

(A26) $$\frac{\partial p_{2}}{\partial p_{1}}=0,\;\frac{\partial p_{2}}{\partial p_{2}}=1,$$

and

(A27) $$\frac{\partial p_{3}}{\partial p_{1}}=-1,\;\frac{\partial p_{3}}{\partial p_{2}}=-1,$$

Substitution gives

(A28) $$g_{ab}\left(p\right)=\frac{\delta_{ab}}{p_{a}}+\frac{1}{p_{3}}$$

Explicitly,

(A29) $$g_{11}=\frac{1}{p_{1}}+\frac{1}{p_{3}}$$

(A30) $$g_{22}=\frac{1}{p_{2}}+\frac{1}{p_{3}}$$

and

(A31) $$g_{12}=g_{21}=\frac{1}{p_{3}}$$

The square-root embedding is

(A32) $$p\mapsto y=\left(2\sqrt{p_{1}},2\sqrt{p_{2}},2\sqrt{p_{3}}\right)$$

This embedding satisfies

(A33) $$y_{1}^{2}+y_{2}^{2}+y_{3}^{2}=4\left(p_{1}+p_{2}+p_{3}\right)=4$$

Therefore, the probability simplex is embedded into the positive octant of a sphere of radius 2.

The Euclidean line element induced by the embedding is

(A34) $$dy_{1}^{2}+dy_{2}^{2}+dy_{3}^{2}=\sum_{i=1}^{3}\frac{1}{p_{i}}dp_{i}^{2}$$

which is the categorical Fisher line element restricted to the simplex. A two-dimensional sphere of radius 2 has Gaussian curvature

(A35) $$K_{simplex}=\frac{1}{2^{2}}=\frac{1}{4}$$

In two dimensions,

(A36) R=2K

Thus, the scalar curvature is

(A37) $$R_{simplex}=\frac{1}{2}$$

For a multinomial sample of size *n*, the Fisher metric is rescaled by *n*,

(A38) $$g_{multinomial}=ng_{categorical}$$

Under constant rescaling of the metric by *n*, scalar curvature rescales by 1/n. Therefore,

(A39) $$R_{multinomial}=\frac{1}{2n}$$

This result is exact for the categorical/simplex Fisher manifold and its multinomial rescaling. In the clinical framework of the main text, it is used as a controlled positive-curvature template for normalized finite-capacity observables. The derivation itself does not imply clinical stability or favorable outcome.

## Appendix C. Graph-Simulation Pseudocode and Computational Protocol

This appendix provides the computational protocol used for the illustrative graph-based diagnostic simulations. The simulation is intended to demonstrate reproducible covariance-based diagnostic proxies. It is not intended to reconstruct exact Fisher scalar curvature unless the corresponding natural-coordinate structure or pullback metric is specified.

### Appendix C.1. Graph Ensemble

The graph is defined as

(A40) G=(V,E,C)

where *V* is the node set, *E* is the edge set, and *C*(*e*) > 0 is the constraint weight assigned to edge e.

The graph-level action is

(A41) $$S_{\lambda }\left(G\right)=\sum_{e\in E}\Phi_{\lambda }(C\left(e\right))$$

with

(A42) $$\Phi_{\lambda }\left(C\left(e\right)\right)=C\left(e\right)+\lambda {\left[C\left(e\right)-\bar{C}\right]}^{2}$$

and

(A43) $$\bar{C}=\frac{1}{M_{E}}\sum_{e\in E}C\left(e\right)$$

The sampling distribution is

(A44) $$P_{\beta ,\lambda }\left(G\right)=\frac{1}{Z(\beta ,\lambda )}\exp\left[-\beta S_{\lambda }(G)\right]$$

### Appendix C.2. Graph Observables

For each graph configuration, the local node-level constraint burden is

(A45) $$c\left(v\right)=\frac{1}{d(v)}\sum_{e\sim v}C(e)$$

The fluctuation-type observables are

(A46) $$O_{1}\left(G\right)=\frac{1}{N}\sum_{v\in V}c(v)$$

and

(A47) $$O_{2}\left(G\right)=\frac{1}{N-1}\sum_{v\in V}{\left[c\left(v\right)-O_{1}\left(G\right)\right]}^{2}$$

The graph-derived fluctuation macrostate is

(A48) $$O_{F}\left(G\right)=\left(O_{1}\left(G\right),\sqrt{O_{2}\left(G\right)}\right)$$

For the compositional sector, two thresholds are defined,

(A49) $$\tau_{1}<\tau_{2}$$

Each edge is assigned to a motif class,

(A50) $$m\left(e\right)=\left\{\begin{array}{c} 1,\;C\left(e\right)<\tau_{1}\\ 2,{\;\tau }_{1}\leq C\left(e\right)<\tau_{2}\\ 3,\;C\left(e\right)\geq \tau_{2} \end{array}\right.$$

The motif fractions are

(A51) $$p_{i}\left(G\right)=\frac{n_{i}(G)}{M_{E}}$$

where

(A52) $$n_{i}\left(G\right)=\left|\left\{e\in E:\;m\left(e\right)=i\right\}\right|$$

The graph-derived compositional macrostate is

(A53) $$O_{C}\left(G\right)=\left(p_{1}\left(G\right),p_{2}\left(G\right),p_{3}\left(G\right)\right)$$

### Appendix C.3. Pseudocode

**Algorithm A1.** Graph-based covariance diagnostic simulation.
**Input:** N, k, β, λ, $\tau_{1}$, $\tau_{2}$, number of Monte Carlo steps, burn-in period, sampling interval, proposal scale, random seed, and regularization parameter ε. **Output:** Retained graph observables, covariance proxies Σ_F_ and Σ_C_, determinants, condition numbers, and stability flags. 1. Initialize an undirected graph G = (V, E, C) with N nodes and mean degree k. 2. Set M_E_ = ∣E∣. 3. Initialize positive edge weights C(e) > 0. 4. Compute the initial graph action Sλ(G). 5. For each Monte Carlo step: - Select one edge e ∈ E. - Propose a local update C(e) → C′(e). - Compute the proposed action Sλ(G′). - Accept the proposal with probability
(A54) $$A\left(G\to G^{\prime }\right)=\min\left\{1,\exp\left[-\beta \left(S_{\lambda }\left(G^{\prime }\right)-S_{\lambda }(G)\right)\right]\right\}$$
6. After burn-in, retain graph configurations at the specified sampling interval. 7. For each retained graph, compute
(A55) $$O_{F}\left(G\right)=\left(O_{1}\left(G\right),\sqrt{O_{2}\left(G\right)}\right)$$
and
(A56) $$O_{C}\left(G\right)=\left(p_{1}\left(G\right),p_{2}\left(G\right),p_{3}\left(G\right)\right)$$
8. Estimate the fluctuation covariance proxy from $\left(O_{1}\left(G\right),\sqrt{O_{2}\left(G\right)}\right)$:
(A57) $$\Sigma_{F}=Cov\left[O_{1}\left(G\right),\sqrt{O_{2}\left(G\right)}\right]$$
9. Estimate the compositional covariance proxy from $\left(p_{1}\left(G\right),p_{2}\left(G\right)\right)$:
(A58) $$\Sigma_{C}=Cov\left[p_{1}\left(G\right),p_{2}\left(G\right)\right]$$
The component $p_{3}\left(G\right)$ is omitted as an independent coordinate because
(A59) $$p_{1}\left(G\right)+p_{2}\left(G\right)+p_{3}\left(G\right)=1\;$$
10. Regularize covariance matrices when needed:
(A60) $$\Sigma_{\varepsilon }=\Sigma +\varepsilon \frac{tr(\Sigma )}{d}I_{d}$$
11. Compute determinant and condition number:
(A61) $$D=\det\left(\Sigma_{\varepsilon }\right)$$ (A62) $$\kappa =\frac{\lambda_{max}\left(\Sigma_{\varepsilon }\right)}{\lambda_{min}\left(\Sigma_{\varepsilon }\right)}$$
12. Mark a grid cell as numerically stable if
(A63) D>0
and
(A64) $$\kappa <\kappa_{max}$$

### Appendix C.4. Baseline Parameters

The baseline simulation used

N = 30, M_E_ = 60, k = 4, (A65)

β ∈ {0.2, 0.5, 1.0, 2.0}, λ ∈ {0.0, 0.5, 1.0, 2.0}, (A66)

τ_1_ = 0.8, τ_2_ = 1.2, (A67)

with 3500 Monte Carlo steps, 1000 burn-in steps, a sampling interval of 25, and 100 retained graph samples per run. Three independent random seeds were used for each (β,λ) grid point.

The covariance regularization parameter was

ε = 10^−6^. (A68)

The numerical outputs are interpreted as covariance-based diagnostic proxies, not as exact Fisher scalar-curvature reconstructions.

## Appendix D. Sensitivity-Analysis Protocol

This appendix describes the sensitivity-analysis protocol used to evaluate whether the graph-based diagnostic quantities were robust under changes in graph size, constraint penalty, heterogeneity penalty, and motif thresholds.

The baseline configuration was

N = 30, M_E_ = 60, k = 4, β = 1.0, λ = 0.5, τ_1_ = 0.8, τ_2_ = 1.2. (A69)

The sensitivity configurations were defined as follows.

**Table A1:** Sensitivity configurations used for graph-based diagnostics.

Configuration	N	M_E_	k	β	λ	τ_1_	τ_2_
Baseline	30	60	4	1.0	0.5	0.8	1.2
Smaller graph	20	40	4	1.0	0.5	0.8	1.2
Larger graph	40	80	4	1.0	0.5	0.8	1.2
Low β	30	60	4	0.2	0.5	0.8	1.2
High β	30	60	4	2.0	0.5	0.8	1.2
High λ	30	60	4	1.0	2.0	0.8	1.2
Wide thresholds	30	60	4	1.0	0.5	0.7	1.3
Narrow thresholds	30	60	4	1.0	0.5	0.9	1.1

For each configuration, three independent random seeds were used. The same Monte Carlo settings were retained across configurations unless otherwise specified. For each run, the following quantities were reported:

(A70) $$D_{F}=\det\left(\Sigma_{F}\right)$$

(A71) $$\kappa_{F}=\frac{\lambda_{max}\left(\Sigma_{F}\right)}{\lambda_{min}\left(\Sigma_{F}\right)}$$

(A72) $$D_{C}=\det\left(\Sigma_{C}\right)$$

(A73) $$\kappa_{C}=\frac{\lambda_{max}\left(\Sigma_{C}\right)}{\lambda_{min}\left(\Sigma_{C}\right)}$$

and

(A74) $${\hat{I}}_{C}=\sum_{i=1}^{3}{\left({\hat{p}}_{i}-\frac{1}{3}\right)}^{2}$$

A diagnostic run was considered numerically stable if the regularized covariance proxy had a positive determinant and a condition number below the pre-specified threshold,

(A75) D>0

(A76) $$\kappa <\kappa_{max}$$

The sensitivity analysis was not designed to test clinical validity. Its purpose was to determine whether the proposed graph-derived observables remain numerically usable under reasonable changes in graph size, constraint parameters, heterogeneity parameters, and motif definitions.

The analysis showed that all tested configurations satisfied the numerical stability criterion. However, condition numbers and compositional imbalance values changed with β, λ, graph size, and motif thresholds. This supports the use of sensitivity reporting in future applications and confirms that graph-derived diagnostics should be treated as implementation-dependent numerical summaries.

## Appendix E. Relation to the Previous Relational–Informational Framework

This appendix clarifies the relationship between the present clinical framework and the previously proposed relational–informational framework for emergent geometry and effective spacetime.

The earlier relational–informational framework introduced an abstract setting in which relational constraints, informational accessibility, and effective geometric structure were linked at a conceptual and mathematical level [11]. In that setting, the focus was on emergent geometry and effective spacetime interpretation.

The present manuscript uses a restricted and clinically oriented adaptation of that idea. No physical spacetime curvature is assumed. No claim is made that clinical states generate gravitational, cosmological, AdS-like, or de Sitter-like geometries. Instead, the relational framework is used only as a way to organize clinical variables, dependencies, constraints, and graph-derived observables before mapping them to statistical manifolds.

The clinical graph is defined as

(A77) G=(V,E,C)

where *V* contains clinical nodes, *E* contains relational edges, and *C*(*e*) > 0 assigns a constraint weight to each edge. In this setting, *C*(*e*) does not represent physical distance. It represents a clinical-relational burden, instability load, resistance, cost, coupling strength, or constraint weight, depending on the application.

The transition from the previous relational framework to the present clinical framework can be summarized as follows.

**Table A2:** Conceptual restriction from relational–informational geometry to clinical statistical geometry.

Previous Relational–Informational Setting	Present Clinical Statistical Setting
Emergent geometry and effective spacetime	Clinical state organization
Relational accessibility and constraint	Clinical dependence, burden, instability, or coupling
Effective geometric interpretation	Fisher-geometric statistical interpretation
Physical or abstract relational systems	Clinical variables, biomarkers, imaging regions, trajectory states
Geometry as emergent relational structure	Geometry as statistical distinguishability
Broad theoretical scope	Methodological clinical hypothesis generation

The key change is that geometry is interpreted here only through Fisher information geometry. Distances, curvature, and metric conditioning refer to statistical distinguishability among probability distributions or graph-derived state ensembles. They do not refer to anatomical distance, physical spacetime, or gravitational curvature.

This restriction is important for clinical interpretation. It prevents conceptual overextension and keeps the framework tied to observable data structures. The graph-based construction provides a reproducible way to map clinical systems into candidate macrostates, but the Fisher-geometric interpretation applies only after a statistical family, coordinate system, and diagnostic procedure have been specified.

The present study therefore uses the previous relational–informational framework as a conceptual motivation, while narrowing its scope to clinical statistical state spaces. The resulting framework is methodological, not metaphysical or physical. Its purpose is to support hypothesis generation and future validation in disease-specific datasets.
